# Secrets behind Protein Sequences: Unveiling the Potential Reasons for Varying Allergenicity Caused by Caseins from Cows, Goats, Camels, and Mares Based on Bioinformatics Analyses

**DOI:** 10.3390/ijms24032481

**Published:** 2023-01-27

**Authors:** Shuai Zhao, Fei Pan, Shengbao Cai, Junjie Yi, Linyan Zhou, Zhijia Liu

**Affiliations:** 1 Faculty of Food Science and Engineering, Kunming University of Science and Technology, Kunming 650500, China; 2 Yunnan Engineering Research Center for Fruit & Vegetable Products, Kunming 650500, China; 3 International Green Food Processing Research and Development Center of Kunming City, Kunming 650500, China; 4 Beijing Engineering and Technology Research Center of Food Additives, Beijing Technology and Business University, Beijing 100048, China

**Keywords:** bioinformatics, casein allergy, linear B-cell epitope, T-cell epitope, allergenic peptides

## Abstract

This study systematically investigated the differences in allergenicity of casein in cow milk (CM), goat milk (GM), camel milk (CAM), and mare milk (MM) from protein structures using bioinformatics. Primary structure sequence analysis reveals high sequence similarity between the *α*-casein of CM and GM, while all allergenic subtypes are likely to have good hydrophilicity and thermal stability. By analyzing linear B-cell epitope, T-cell epitope, and allergenic peptides, the strongest casein allergenicity is observed for CM, followed by GM, and the casein of MM has the weakest allergenicity. Meanwhile, 7, 9, and 16 similar or identical amino acid fragments in linear B-cell epitopes, T-cell epitopes, and allergenic peptides, respectively, were observed in different milks. Among these, the same T-cell epitope FLGAEVQNQ was shared by κ-CN in all four different species’ milk. Epitope results may provide targets of allergenic fragments for reducing milk allergenicity through physical or/and chemical methods. This study explained the underlying secrets for the high allergenicity of CM to some extent from the perspective of casein and provided new insights for the dairy industry to reduce milk allergy. Furthermore, it provides a new idea and method for comparing the allergenicity of homologous proteins from different species.

## 1. Introduction

Food allergy is a global problem; moreover, the number of people with allergies and types of food allergens is increasing every day. The eight major food allergens are eggs, fish, milk, tree nuts, peanuts, wheat, shellfish, and soybean [1]. Cow milk (CM), as the most popular and commonly consumed dairy product, accompanies our daily life; in addition, it is also the most prone to induce allergies in the most common dairy products [2]. Around 2–3% of children under the age of two are allergic to CM [1]; for these young children, we can only either reduce the allergenicity of allergens in CM by physical and/or chemical methods [3,4] or instead find alternative milk sources [5]. Goat milk (GM), camel milk (CAM), and mare milk (MM) are reported to be less allergenic than CM in children with a CM allergy. In addition, they are considered high-quality raw milk sources that can serve as an alternative to CM [5,6]. However, CM comprises 82.4% of the world’s fresh milk and remains the most widespread source of dairy products worldwide [2]. Therefore, determining the reason behind the higher allergenicity of CM, in comparison with other dairy products, is still an urgent issue to consider.

The major allergens in CM are represented by whey protein and casein, whereby the latter accounts for around 80% [7]. In the total proteins of GM, CAM, and MM, the casein content is also the highest [6,8,9]. Casein includes four major subtypes: α_S1_-casein (α_S1_-CN), α_S2_-casein (α_S2_-CN), β-casein (β-CN), and κ-casein (κ-CN) [7]. Among these subtypes, α-CN is the most allergenic protein, followed by κ-CN [10]. Moreover, the casein in CM is highly susceptible to hydrolysis by pepsin [11]. In addition, the polypeptides produced after proteolysis may have functional effects on the human body [12] or can cause, in combination with immunoglobulin E, allergic reactions [13]. The composition and sequence of amino acids determine the higher structure of proteins, which, in turn, entails the biological function of proteins, including allergenicity. Certain previous studies have found that the allergenicity of GM, CAM, and MM is significantly lower than that of CM [5,6], but no report has clearly determined the reason, especially from the perspective of the difference in amino acid composition and the sequence of caseins. A previous study has used bioinformatics to compare the differences in amino acid compositions and the sequences of lactoferrin from different mammals in order to elucidate the similarity of the primary and higher structure of lactoferrin [14]. With the advancement of protein sequence analysis technology [15], the primary structures of many proteins have been resolved. Much of the current research has been based on bioinformatics technology. Such studies have discovered novel functional proteins [16,17], active peptides (hypoglycemic, hypotensive, and antibacterial) [12,18], and adverse hydrolysates (toxicity and allergenicity) [19,20]. Databases with experimental data of allergenic proteins have also been gradually obtained (e.g., DNASTAR Protean, BepiPred1.0, ABCpred, IEDB, and NetMHCIIpan-4.0 server), which thus enables the study on the causes of the allergenicity of proteins through bioinformatics. Furthermore, using bioinformatics can rapidly and accurately predict and screen allergenic proteins, including allergenic peptides. Their accuracies may be not as rigorous as traditional experiments in the past, but the results are now becoming increasingly more accurate with the update of these databases, as well as the further optimizations being conducted with respect to the algorithms.

Therefore, a “bottom-up” approach was applied in this study in order to discover the information behind the protein sequence for unveiling the reason of casein allergy in different species. This was achieved by analyzing the: amino acid sequence, physicochemical properties, secondary structure, linear B-cell epitope, allergenic peptide, and T-cell epitope. Furthermore, a study on the number of linear B-cell epitopes, T-cell epitopes, and allergenic peptides in the protein could further identify the allergenicity strength of protein. The results of this study may provide certain new insights and strategies for the reduction of milk allergy cases in the dairy industry. It may also supply a new model for the screening and predicting of other foodborne protein allergies.

## 2. Results and Discussion

### 2.1. Analysis of the Composition and Content of Casein in Human Milk (HM), CM, GM, CAM, and MM

In the present study, the allergenicity strength of protein was inferred by using the number of linear B-cell epitopes, allergenic peptides, and T-cell epitopes. In order to achieve this purpose, the content of casein in the different kinds of milk was initially studied. Thereinto, we will also add HM—which was considered to possess no allergenicity—to the discussion and, from the perspective of casein composition and content, explore the casein difference between HM and the abovementioned kinds of milk. The contents of casein and its four different subtypes in HM, CM, GM, CAM, and MM are summarized in Table 1, respectively. CM possessed the highest content of casein, followed by GM, while HM had the lowest content of casein. Among the four subtypes of caseins in HM, GM, CAM, and MM, the content of β-CN was found to be the highest in all of them. Meanwhile, with respect to CM, the content of α_S1_-CN was found to be the highest. Conversely, the lowest subtype in HM, CM, and MM was found to be α_S2_-CN. Additionally, α_s1_-CN and κ-CN corresponded to the lowest subtype in GM and CAM, respectively. The study performed by Natale et al. showed that α_s1_-CN, α_s2_-CN, and κ-CN were the major allergenic subtypes of casein in CM [10]. As shown in Table 1, the α-CN contents (α_s1_-CN and α_s2_-CN) of CM, GM, CAM, and MM were around 53.57%, 24.80%, 31.50%, and 19.26%, respectively. The total contents of the most predominant allergenic subtypes (α-CN and κ-CN) in CM, GM, CAM, and MM were about 65.87%, 45.20%, 35.00%, and 21.04%, respectively. In addition, previous studies have reported that CM was the most allergenic milk when compared with GM, CAM, and MM [5,6]. Therefore, the high content of casein in CM, especially the high contents of allergenic subtypes (α-CN and κ-CN), may be one of the most important reasons for its high allergenicity. Furthermore, we found that CAM and MM possessed a similar casein composition to HM. Therefore, this may also be one of underlying reasons for their lower allergenicity [6,9].

Notably, accurately distinguishing the allergenicity of casein from different species may be difficult when only analyzing the contents of the main allergenic subtypes. As shown in Table 1, the contents of α-CN and κ-CN in CAM were higher than their counterparts in MM. However, a previous study has reported that CAM possessed less allergenicity than MM [23]. Therefore, the allergy research of the different species of milks should not only consider the content of the overall allergenic subtypes, but also analyze the allergenicity characteristics of individually allergenic subtypes. As mentioned above, the composition and sequence of amino acids determine the higher structure of protein, which may, in turn, determine the biological function of protein, including allergenicity. Thus, further analyses of the amino acid sequences and secondary structures of casein may be the key point by which to unveil the secret behind the allergenic differences of caseins from different milk sources.

### 2.2. Sequence Similarity Analysis of α_S1_-CN, α_S2_-CN and κ-CN in CM, GM, CAM, and MM

**Figure 1:**
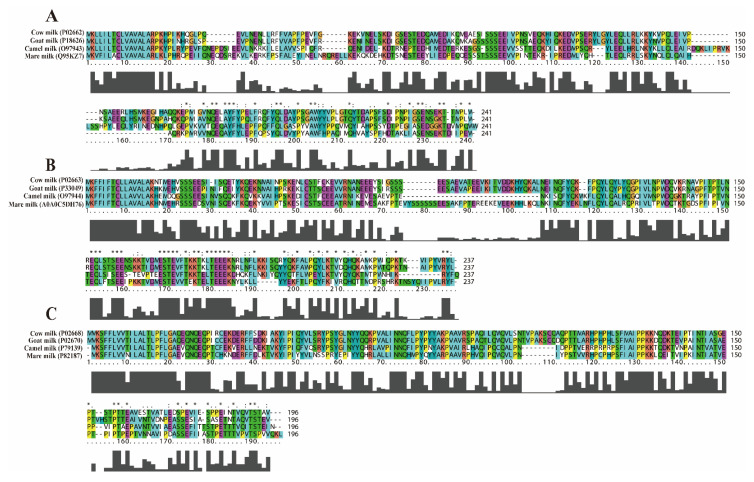
The alignment of amino acid sequences of caseins, as derived from the four different species’ milk. The allergenic subtypes of CM were used as a template, where panels (**A**–**C**) show the aligned amino acid sequence of α_S1_-CN, α_S2_-CN, and κ-CN, respectively. The height of the bar plots under each sequence alignment represents the number of identical amino acids from the four different species’ milk (whereby a maximum was four and the minimum was zero). The number marked on the left is the UniProtKB database accession number.

The allergenicity of protein is considered to be closely related to its amino acid sequence. In this work, Clustalx software [24] was utilized in order to conduct a sequence comparison study for the purpose of understanding the differences in the amino acid sequences of allergenic subtypes in the four different species’ milk. The most predominant allergenic subtypes of CM (α_S1_-CN, α_S2_-CN, and κ-CN) were used as templates in order to compare their sequence similarity with the counterpart of three other milk proteins. The results of this are shown in Figure 1. In regard to α_S1_-CN—when the amino sequence of GM, CAM, and MM was aligned to that of CM—it was found the sequence similarity of GM, CAM, and MM was 89.72%, 46.96%, and 37.38%, respectively, when compared with CM. In further relation to α_S2_-CN, the sequence similarity of these three different species’ milk was found to be 90.13%, 54.05%, and 58.01%, respectively, compared with CM. Regarding the κ-CN, when compared with CM, the amino sequence similarity was 59.47%, 55.36%, and 56.32% for GM, CAM, and MM, respectively. This result demonstrated that GM and CM possess a higher homology in α-CN, while GM, CAM, and MM possessed a low sequence similarity with CM with respect to κ-CN. Restani et al. also reported that the proteins of CAM and MM possessed lower homologies than CM, which was determined by comparing the similarities of 3D protein conformations with values of 60% and 62.4%, respectively. Meanwhile, the protein of GM possessed a higher homology than CM with a value of 87.6% [25]. Given that the composition and sequence of amino acids most likely lead to the different functions and allergenicity of CM, which is the strongest among the four different species’ milk [5,6], the abovementioned results indicate that the composition and sequence of amino acids of the four milk proteins most likely also play an important role in their allergenicity. In addition to this, we also compared the HM sequence similarity of α_S1_-CN (Figure S1A) and κ-CN (Figure S1B) with the different species’ milk in order to discover the similarities of allergenic subtypes of casein between HM and the above milk. The amino sequences of CM, GM, CAM, and MM were aligned to HM, with respect to α_S1_-CN. Through conducting this, it was found that the sequence similarity of CM, GM, CAM, and MM was 34.59%, 34.05%, 38.92%, and 44.86%, respectively, when compared with HM. Regarding κ-CN, when compared with HM, the amino sequence similarity was 47.25%, 50.55%, 56.04%, and 66.48% for CM, GM, CAM, and MM, respectively. When compared to CM and GM, the two allergenic subtypes (i.e., α_S1_-CN and κ-CN) of CAM and MM were found to be evidently more similar to HM. Therefore, this could be one of the underlying reasons for the lower allergenicity of CAM and MM. In order to further explore the role of amino acid sequence differences on the allergenicity of the allergenic proteins from the four different milk species, the linear B-cell epitope and T-cell epitope require further analysis via the method of prediction.

### 2.3. Analyses of Physicochemical Property

Before allergenicity analysis, the physicochemical properties of allergenic proteins in the four different species’ milk were first analyzed. The hydrophilia and hydrophobicity of α_S1_-CN, α_S2_-CN, and κ-CN were investigated via bioinformatics in order to explore the hydrolysis susceptibility of the four different species’ milk. In CM, GM, CAM, and MM, as shown in Table 2, the grand average of the hydropathicity (GRAVY) values of α_S1_-CN, α_S2_-CN, and κ-CN were all less than zero, which was predicted and analyzed via using PortParam [26]. The GRAVY value is usually used to evaluate the hydrophilia and hydrophobicity of proteins. Moreover, the GRAVY value ranges between −2 and 2, where a negative or positive value indicates that the protein possesses good hydrophilia or hydrophobicity, respectively. When there is an average value of GRAVY with respect to soluble proteins, it was considered to be −0.4 [27]. The results showed that all the α_S1_-CN, α_S2_-CN, and κ-CN of CM, GM, CAM, and MM possessed good hydrophilia, and all the α_S1_-CN and α_S2_-CN of the four different species’ milk were considered soluble proteins (Table 1). Meanwhile, the proportion of the polar groups of α_S1_-CN, α_S2_-CN, and κ-CN of CM, GM, CAM, and MM was evidently higher than that of the non-polar groups (Figure S2). Therefore, they can be easily digested upon entering the GI tract. In addition, casein is very sensitive to hydrolysis by pepsin [11], and thus it is considered an excellent source of amino acids [9].

In a protein, the aliphatic index (AI) represents the proportion of alanine, valine, isoleucine, and leucine in the relative volume of the aliphatic side chain. Moreover, it can be used to estimate the thermal stability of the protein [28]. With respect to this, Rehman et al. found that when the AI value was higher than 65, the protein possessed good thermal stability [29]. As shown in Table 2, the AI values of the α_S2_-CN in relation to the four different species’ milk were lower than that of the α_S1_-CN and κ-CN. However, all the AI values were higher than 65, which entails that the α_S1_-CN, α_S2_-CN, and κ-CN of the four different species possessed good thermal stability. Usually, the milk in question was pasteurized before drinking; further, the pasteurization temperature was 65–85 °C [4], which may alter the structure of proteins (e.g., heating causes the protein to unfold and expose more hydrophobic regions). As such, this fact resulted in a reduction in bioactivities or the allergenicity of proteins [11]. High temperature was considered to be a common method by which to reduce allergenicity in milk. However, the allergenic proteins possess good thermal stability (Table 2). This implies that common sterilization, called pasteurization, at temperatures of 65–85 °C will have no, or a minimal, effect on the structure of these allergenic proteins. Therefore, it may be required to increase the temperature during pasteurization in order to reduce their allergenicity by heating. However, this may lead to a loss of many nutrients. Moreover, Gomaa et al. reported that the casein allergenicity recovery of cookies made by mixing casein with flour was 49%, after 25 min of baking at 105 °C in the cookie center [30], which could provide support for this hypothesis.

According to the prediction of physicochemical properties, the current study found that the allergenic subtypes of casein in the four different species’ milk possessed good hydrophilicity and thermal stability, albeit with some differences. However, these findings cannot be explained by the underlying reasons regarding the differences in protein allergenicity in the four different species’ milk as, via an analysis of the physicochemical properties, the differences were marginal. Therefore, the relationship between allergenicity and protein structure needs to be further explored through a prediction of the linear B-cell epitope, secondary structure, and T-cell epitope of proteins.

### 2.4. The Prediction of the Secondary Structure, the Linear B-Cell Epitope of Proteins, and the Screening of Allergenic Peptides

**Figure 2:**
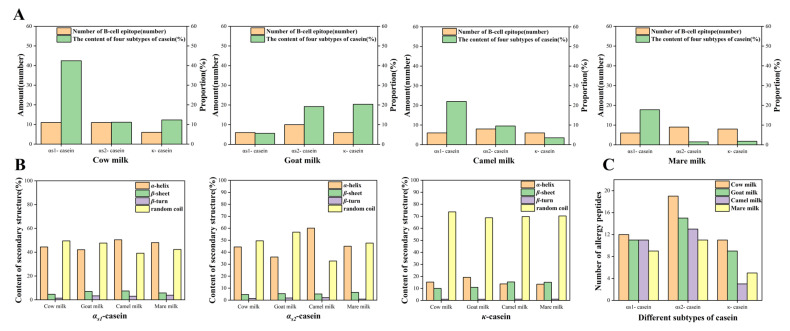
The predicted results of the linear B-cell epitopes (**A**); the secondary structures (**B**); and the allergenic peptides (**C**) of allergenic subtypes, respectively, in CM, GM, CAM, and MM. The panels in (**A**) represent the number of linear B-cell epitopes, as well as the corresponding allergenic subtype proteins that occupy the casein content (the left and right of the ordinate represent the amount of casein and the proportion, respectively). The panels in (**B**) represent the contents of the α-helix, β-sheet, β-turn, and the random coil of allergenic subtypes. Lastly, the panel in (**C**) represents the number of allergenic peptides.

Antigenic epitopes are the immunological basis of allergenic molecules through which they trigger food allergic reactions by their binding through antibodies [31]. Among them, the linear epitope is composed of continuous amino acids and can be identified by the primary structure of protein, while the conformational epitope consists of spatially adjacent, discontinuous amino acid residues and is related to the three-dimensional (3D) structure of proteins [31]. If different databases are adopted for the prediction of linear B-cell epitopes, a different result may be obtained due to the variations present in the different analysis methods with respect to each database. In order to better understand the linear B-cell epitope, two different web servers (BepiPred 2.0 server [32] and IEDB server [33]) were used for the prediction of the linear B-cell epitope in this study. With respect to this, the prediction results are shown in Table S2. The final prediction results of the linear B-cell epitope were generally, and consistently, obtained by two servers, which indicates that the prediction was reasonable and accurate to some extent. The sequence fragments of polypeptides and the locations of the linear B-cell epitope are summarized in Figure S3. Moreover, the linear B-cell epitope consists of multiple amino acids, which were arranged either as continuous or discontinuous within a certain 3D structure [31]. The linear B-cell epitopes of α_S1_-CN, α_S2_-CN, and κ-CN were evenly distributed in the whole sequence with numerous instances of long peptides (Figure S3). At the same time, the predicted results of the linear B-cell epitopes of the allergenic subtypes in CM were considered to be highly similar to the reported corresponding epitope results [34,35,36]. This was of particular relevance for the linear B-cell epitope predicted results of κ-CN. Therefore, the results of the epitope comparisons show that the prediction results may possess a high accuracy. As shown in Figure 2A, the number of recognized polypeptide fragments of linear B-cell epitopes (NRPFLB-cellE), with respect to α_S1_-CN and α_S2_-CN in CM was found to be the highest (11 and 11, respectively), when compared with their counterparts in GM, CAM, and MM. This finding is consistent with the results of a previous study on the NRPFLB-CellE of α_S1_-CN and α_S2_-CN, in comparison between CM and GM [37]. Meanwhile, the NRPFLB-cellE of κ-CN with respect to CM, GM, CAM, and MM possessed a number of 6, 6, 6, and 8, respectively. A previous study found that α-CN possessed a higher allergenicity than that of κ-CN in CM [10]. Moreover, the aforementioned results could explain well the potential reasons for the higher allergenicity of α-CN (α_s1_-CN and α_s2_-CN) when in comparison with the κ-CN in CM. Notably, MM possessed the highest NRPFLB-cellE in regard to κ-CN. Having said this, MM has been proven to be less allergenic than CM [6]. In addition, this fact may be explained by the low content of κ-CN in MM. Therefore, the allergenicity of protein may be closely related to NRPFLB-cellE and its content. From the perspective of casein allergenicity analysis, the NRPFLB-CellE results may explain the reason behind the higher allergenicity of CM than the allergenicity of GM and CAM, as well as of MM (though to a lesser extent).

As shown in Figure 2A, GM not only possessed higher contents of allergenic subtypes (i.e., α-CN and κ-CN), but also possessed higher NRPFLB-cellE when compared with its counterparts in CAM or MM. When comparing CAM and MM, the content of α_s2_-CN and κ-CN in MM was found to be very low [6]. In addition, the α_s1_-CN content of MM was also lower than those found in CAM, but their NRPFLB-cellE was the same. Therefore, the allergenicity of CAM from the perspective of casein allergenicity may be slightly stronger than that of MM. However, a previous study reported that CAM, which shares a similar protein composition to HM, was the least allergenic milk [38]. This difference in results may be due to the fact that the allergenic proteins in milk include not only casein, but also whey protein. Moreover, β-Lactoglobulin (β-lg), which is a type of whey protein, has also been regarded as the main allergenic protein of milk; however, this compound is not contained in CAM [3,38]. Therefore, as a whole, the allergenicity of CAM may be slightly weaker than that of MM. With respect to this, Villa et al. reported that people who are allergic to CM also possess a high cross-reactivity to GM but not to CAM or MM [2]. In addition, a previous study suggested that CAM can be considered a good substitute for children who are allergic to CM and GM [5]. Therefore, the results of the current work clearly demonstrate that the analyses of NRPFLB-cellE, and of its content regarding allergenic subtypes, appear to be a reliable and accurate method by which to predict the allergenicity of food. Furthermore, such a method also aids in unveiling the underlying reasons for allergenicity.

Seven similar, or identical, linear B-cell epitopes were found from the allergenic subtypes of casein with respect to the four different species’ milk (Table S3). In regard to this, they appeared to mainly exist in CM and GM (6). Through conducting the sequence similarity analysis, we found a high degree of similarity between the α-CN of CM and GM. Therefore, this factor could be the reason behind their many similar linear B-cell epitopes. Interestingly, with respect to κ-CN, CAM and MM also possessed a similar linear B-cell epitope, VQNQEQPTC, while the κ-CN similarity between CAM and MM was also slightly higher. The destruction of allergenic fragments or epitopes when using physical or chemical methods (i.e., high pressure, high temperature, and enzymatic hydrolysis), is considered effective in terms of reducing the allergenicity of these compounds. Therefore, the common linear B-cell epitopes obtained via prediction of the allergenic subtypes with respect to the four different species’ milk in this study, may provide allergenic fragments that could assist with the aim of reducing the abovementioned milks’ allergenicity—which would be achieved via the use of physical and/or chemical methods.

The prediction of epitopes is closely related to the properties of proteins, which is especially the case for the secondary structure characteristics. Meanwhile, the α-helix and β-sheet structures are not so easily deformed and are difficult to combine with antibodies in order to form epitopes [32]. Therefore, the β-turn and random coil are considered to more easily bind to antibodies as epitopes. As shown in Table S4 and Figure 2B, the α_s2_-CN of MM possessed the highest contents of β-turn and of a random coil. Moreover, the κ-CN of CM contained the highest contents of β-turn and of a random coil. Meanwhile, no obvious difference in α_s1_-CN was observed between CM and GM. In addition, the contents of β-turns and random coils in the four kinds of milk were inconsistent with respect to their quantity of NRPFLB-cellE. Thus, when observing the distribution of α-helix, β-sheet, β-turn, and random coil in the proteins, it is necessary to further study the relationship between the proteins’ secondary structure and linear B-cell epitope. As shown in Figure S4, the α-helix and β-sheet of the α_s1_-CN in CM were widely distributed within a short sequence, which resulted in β-turns and random coils being present as long sequences with numerous allergenic fragments. The three other different species’ milk engendered the opposite result. In regard to this, the long secondary structure fragment can easily form epitopes, which may be the reason for the high content of NRPFLB-cellE. Similarly, the α_s2_-CN in CM and GM possessed longer sequences of β-turns and random coils than those found in CAM and MM. In contrast to the aforementioned prediction results, a small degree of difference in the amount of NRPFLB-cellE was found between CM and GM. Regarding κ-CN, the β-sheet in MM divided the random coil into a number of uniform long sequences, while none of the three other different species’ milk possessed such long sequence segments to the degree found in MM. This result indicates that the number of NRPFLB-cellE with respect to κ-CN in MM was the highest, which was contrary to the aforementioned prediction (Figure 2A). Therefore, the accuracy of the linear B-cell epitope prediction was demonstrated well by the secondary structure analysis.

Proteins, including allergenic proteins, are hydrolyzed into peptides in the GI tract. Moreover, in regard to this, peptides that possess allergic fragments, or structures, are also allergenic. Therefore, in this study, the allergenic proteins were further subjected to a simulated GI hydrolysis; further, the peptides were also predicted for, with respect to allergenicity. Meanwhile, good water solubility was the key to peptide digestion and metabolism in vivo [39]. Further, the final allergenic peptides were obtained by predicting the degree of allergenicity and water solubility, as shown in Table S5. In terms of the sequence structure of peptides, most of the allergenic peptides that followed simulated proteolysis were peptides below ten, which was mainly observed in the dipeptides to tetrapeptides. As shown in Figure 2C, the number of allergenic peptides of α-CN (α_s1_-CN and α_s2_-CN) was evidently higher than that of κ-CN in all four of the different species’ milk. This finding is similar to the results of certain literature reports [10] and linear B-cell epitope predictions. Indeed, after proteolysis was simulated, the number of allergenic peptides, as well as the proportion of allergenic/enzyme digestion peptides with respect to α_S1_-CN, α_S2_-CN, and κ-CN in CM were found to be the highest (Table 3). Regarding the α_S1_-CN, α_S2_-CN, and κ-CN in GM—with the exception of the proportion of allergenic/enzyme digestion peptides of κ-CN—the number of allergenic peptides and the proportion of allergenic/enzyme digestion peptides closely followed those found in CM, as is shown in Table 3. With respect to CAM and MM, when comparing the number of allergenic peptides and the proportion of allergenic/enzyme digestion peptides, it was concluded that the α-CN in CAM was higher, while the quantity of κ-CN in MM was more (Table 3). However, the content and number of allergenic peptides, as well as the proportion of allergenic/enzyme digestion peptides with respect to κ-CN were all found to be very low in MM. Therefore, the results of allergenicity regarding the four different species’ milk, from the perspective of casein allergenicity, indicated that the allergenic peptides are consistent with the results of linear B-cell epitope. Furthermore, the present study found that the linear B-cell epitopes may be destroyed by GI digestion, as well as the fact that the number of allergenic peptides produced by the hydrolysis of epitopes was the most prominent regarding the total allergenic peptides, especially in the case of the quantity of casein in CM (Table 3). Therefore, the hydrolyzation of linear B-cell epitopes may be a feasible approach by which to reduce allergenicity. Notably, among all the predicted allergenic peptides, a total of 16 allergenic peptides were derived from more than an allergenic protein alone (Table S6). Among these, the same allergenic peptide sequences were generally found to be between 2 and 4 in length. Meanwhile, the allergenic peptides, MK and EK, were widely present in the hydrolysates of the allergenic subtypes of casein with respect to the four different species’ milk. Furthermore, tetrapeptides were considered to host an IgE binding ability [40]; thus, the allergenicity of dipeptides was predicted based on database analysis [41]. Therefore, di-tetrapeptides can be considered potential allergenic peptides. In addition, CM and GM possess many of the same allergenic peptides (14), which may be related to their high sequence similarity.

In this study, the prediction of linear B-cell epitopes and the allergenic peptide analysis of allergenic protein (e.g., in this case, casein) can clearly aid with identifying and analyzing the strength of the allergenicity of food protein, as well as to help unveil the underlying reasons for allergenicity. At the same time, the consistency of the predicted results in conjunction with those reported in the literature [2,5,10], illustrates the feasibility of using this study method in order to probe the allergenicity differences in proteins from investigating their primary structure. In the processing of allergens in the body, peptide fragments containing T-cell epitopes are provided via antigen-presenting cells in conjunction with the major histocompatibility complex (MHC) class II molecular on the surface of cells [42]. Therefore, the recognition of a T-cell epitope was found via the initiation of a sensitization/allergic cascade. Moreover, the prediction of T-cell linear epitopes also develops from the investigation of the protein’s primary structure.

### 2.5. Prediction of T-Cell Epitope of Proteins

In regard to food allergy, T-cell epitopes are the short segments of the allergen, which typically consist of 12–20 contiguous amino acids [31]. Furthermore, T-cell epitopes will form a T-cell epitope–MHC class II molecular complex with an MHC class II [31]. With respect to this, the NetMHCIIpan-4.0 server [43] can use artificial neural networks (ANNs) to predict whether peptides can bind to MHC II classes in order to form epitopes. In conducting the predictions of T-cell epitopes with respect to the four different species’ milk, the number of κ-CN was found to be greater than the individual instances of α_S1_-CN or α_S2_-CN (Table S7). However, the number of the T-cell epitopes of the total α-CN (i.e., α_S1_-CN and α_S2_-CN) was still found to be higher than that of κ-CN, which also indicates that α-CN may be more allergenic than κ-CN. This result is consistent with the findings of the literature [10], as well as with the results of the linear B-cell epitope prediction and the allergenic peptide analysis. In this study, in a comparison regarding the three allergenic subtypes of casein with respect to the four different species’ milk, it was found that the casein in CM contained the highest number of T-cell epitopes, followed by the number of caseins in GM. Moreover, CM and GM possessed the same fragments of T-cell epitopes in α-CN and κ-CN (Table 4). Further, the high sequence similarity between CM and GM may result in them possessing the same degree of T-cell epitope fragments and may also be one of the reasons for the similar allergenicity of CM and GM. In a comparison of CAM and MM, the number of T-cell epitopes of α_S1_-CN in CAM was found to be evidently lower than that found in MM, while CAM possessed a higher content of α_s1_-CN. Meanwhile, the number of T-cell epitopes of α_S2_-CN or κ-CN in CAM and MM was found to be higher than that of α_S1_-CN. Meanwhile, the α_S2_-CN and κ-CN contents in MM were extremely low. Therefore, similar to the linear B-cell epitope results, the overall allergenicity of CAM from the perspective of casein allergenicity may be slightly stronger than that of MM. Notably, the κ-CN of all four different species’ milk shared an identical T-cell epitope, that is, FLGAEVQNQ (Table 4). Furthermore, it may also be the main target for reducing the allergenicity of the four different species’ milk. In summary, the results of the T-cell epitope prediction of allergenicity were consistent with those of the linear B-cell epitope and its allergenic peptides. Furthermore, the present study found that the combination of linear B-cell epitopes and T-cell epitopes may be useful in terms of measuring the strength of casein allergenicity in the four different species’ milk. 

### 2.6. Analysis of the Method Limitations 

Although the difference in allergenicity that was caused by the same proteins that are derived from the four different species’ milk was systematically described in this study, the analysis for allergenicity—which was achieved through investigating the homologous foodborne proteins from the different species—may possess certain errors due to the small sample size (12 samples) adopted. However, the allergenicity analysis that was conducted via various methods in this study indicated a consistent result, which, by itself, demonstrates the reliability of the results obtained in this study. Due to the fact that this study was based on the three potential allergenic subtypes of caseins from the four different species’ milk and due to the fact that it is still a challenge to isolate and purify the potential allergenic subtypes α_s1_-, α_s2_-CN from caseins, the hydrolysis of these three subtypes in a GI digestion simulation has to be conducted in silico by using the available sequence of these subtypes in order to replace in vitro experiments. It is undeniable that certain deviations are present between the in silico and in vitro hydrolyses. However, investigations regarding the hydrolyzed degree of protein in silico and in vitro found that the in silico experiment better simulated the hydrolysis of protein, thus rendering it highly reliable [44]. Furthermore, the accuracy of the prediction for the linear B-cell epitope, T-cell epitope, and allergenic peptides, as based on the database, will be improved with the further upgrade of the database. 

## 3. Material and Methods

### 3.1. Database and Computational Software

The primary structure of α_S1_-CN (accession number of CM: P02662; of GM: P18626; CAM: O97943; of MM: Q95KZ7; of HM: P47710), α_S2_-CN (accession number of CM: P02663; GM: P33049; of CAM: O97944; of MM: A0A0C5DH76;), and κ-CN (accession number of CM: P02668; of GM: P02670; of CAM: P79139; of MM: P82187; of HM: P07498) were downloaded from UniProtKB database (https://www.uniprot.org/) (accessed on 4 May 2022). In addition, Clustalx software (Dublin, UK) [24]; the PSIPRED web server (London, UK) [45]; the NPS SOPMA web server (Lyon, France) [46]; the BepiPred 2.0 web server (Copenhagen, Denmark) [32]; the IEDB web server (La Jolla, USA) [33]; NetMHCIIpan-4.0 server (Copenhagen, Denmark) [43]; the Innovagen (Innovagen AB, Sweden), AllerTOP v 2.0 online server (Sofia, Bulgaria) [19]; and the ExPASy web server (PortParam and Expasy PeptideCutter) [26] were all used in order to perform the bioinformatics analysis.

### 3.2. Compositions and Contents of Caseins in CM, GM, CAM, and MM

According to the literature summary, the contents of the total casein, α_S1_-CN, α_S2_-CN, β-CN, and κ-CN in CM, GM, CAM, MM, and HM were collected [6,8,9,21,22], as shown in Table 1.

### 3.3. Prediction of Property and Structure of α_S1_-CN, α_S2_-CN, and κ-CN

The amino acid sequences of α_S1_-CN, α_S2_-CN, and κ-CN in HM, CM, GM, CAM, and MM were all searched in the UniProtKB database (https://www.uniprot.org/) (accessed on 4 May 2022) (Figure 1 and Figure S1). Furthermore, their amino acid sequences were compared and analyzed using Clustalx software [24]. Moreover, the PortParam web server [26] was used to analyze the physicochemical properties of these proteins. The PSIPRED web server [45] and the NPS SOPMA web server [46] were utilized in order to analyze the polarity and distribution, as well as the secondary structure and distribution of the proteins.

### 3.4. Allergenicity Definition

In this study, the allergenicity of the different kinds of milk and their subsequent comparisons were studied and investigated based on bioinformatics analysis. Furthermore, the allergenicity of protein was defined by the number of linear B-cell epitopes, T-cell epitopes, and the allergenic peptides in the protein.

### 3.5. Prediction of Linear B-Cell Epitopes Regarding α_S1_-CN, α_S2_-CN, and κ-CN

The linear B-cell epitope of proteins was predicted using the BepiPred 2.0 web server [32] and the IEDB web server [33]. The scoring threshold of the BepiPred 2.0 web server epitope assignment was set as 0.35, and IEDB web server was used to predict the linear B-cell epitope. The intersection of the linear B-cell epitope analysis in the BepiPred 2.0 web server and the IEDB web server was extracted in order to obtain the final linear B-cell epitope.

### 3.6. Prediction of T-Cell Epitope of α_S1_-CN, α_S2_-CN, and κ-CN

The identification of T-cell epitopes was focused upon the screening of peptide fragments bound to MHC class II, where the linear T-cell epitopes were predicted by the NetMHCIIpan-4.0 server [43]. The HLA-DQ binding epitope was predicted by the NetMHCII-4.0 server and set to less than 2%, as well as less than 10% for the strong binder and weak binder, respectively. The predicted peptide length was set to 15 amino acids. Moreover, the NetMHCIIpan-4.0 server, through using ANNs, predicted the peptide binding to any type of MHC II molecule in the known sequence [43].

### 3.7. Hydrolysis of α_S1_-CN, α_S2_-CN and κ-CN by In Silico

The program Expasy PeptideCutter [26] was utilized in order to simulate, via GI tract enzymes, the hydrolysis of α_S1_-CN, α_S2_-CN, and κ-CN. The protein hydrolysis was performed by the use of three kinds of enzymes: pepsin (EC 3.4.23.1), trypsin (EC 3.4.21.4), and chymotrypsin (EC 3.4.21.1) [39]. With respect to this, peptides with dipeptides and above were collected for the purposes of further prediction and analysis.

### 3.8. Prediction of Solubility and the Allergies of Peptides 

The peptide property calculator in the Proteomics tools in Innovagen (Innovagen AB, Lund, Sweden) was used to predict the solubility of polypeptides, which was available at http://www.innovagen.com/proteomics-tools (accessed on 20 May 2022). In addition, the allergenic peptides were predicted using the AllerTOP v. 2.0 online server [19]. However, the allergenic peptide predictions were identified by aligning the target peptide and the reported allergenic protein by a self-cross-covariance (ACC) [19]. Moreover, allergenic peptides are peptides that are resistant to digestion and can bind to the IgE Fab in the GI tract. Thus, they may trigger an allergic reaction.

### 3.9. Statistical Analysis

The results are expressed as the mean ± standard deviation (S.D.) (*n* = 3). In addition, a one-way ANOVA in conjunction with a Tukey test was applied in order to evaluate the significant differences (*p* < 0.05), which was achieved by using Origin 8.5 software (Northampton, MA, USA).

## 4. Conclusions

In this study, a rapid method for screening and comparing the allergenicity of homologous proteins from different species was developed by using bioinformatics technology. We investigated the sequence similarity, physicochemical properties, and allergenicity regarding the protein structures of α_S1_-CN, α_S2_-CN, and κ-CN in the four different species’ milk. The results showed that the allergenic subtypes in the four different species’ milk may all possess good hydrophilicity and thermal stability. In terms of casein composition and sequence alignment, we found that CAM and MM were considered to be more similar to HM. In addition, CM and GM possessed a high sequence similarity and were accompanied by similar properties, including allergenicity. The results of the linear B-cell epitope, allergenic peptides, and T-cell epitope analysis revealed that the strongest allergenicity—from the perspective of casein allergenicity—was observed for the casein in CM, followed by the casein in GM, and, finally, the casein in MM possessed the weakest allergenicity. Notably, the results obtained in this study were reliable when compared with previous reports.

In addition, certain amounts of similar amino acid fragments of linear B-cell epitopes and T-cell epitopes were found in the casein in CM and GM. In addition, the same T-cell epitope, FLGAEVQNQ, was shared by κ-CN in all four of the different species’ milk. The results of this study may provide certain new insights with respect to the aim of reducing milk allergies in the dairy industry. Moreover, the completion of this study provides a more economical and convenient method for the allergenicity difference analysis regarding foodborne proteins.

## Figures and Tables

**Table 1:** The proportions of casein, α_S1_-casein (α_S1_-CN), α_S2_-casein (α_S2_-CN), β-casein (β-CN), and κ-casein (κ-CN) in: human milk (HM), cow milk (CM), goat milk (GM), camel milk (CAM), and mare milk (MM).

Protein	Human ^a,b^	Cow ^c^	Goat ^d^	Camel ^e^	Mare ^c^
Casein (%) *	40.00	80.00	64.52	52.00	55.00
α_s1_-casein (%) #	13.79	42.46	5.60	22.00	17.78
α_s2_-casein (%) #	-	11.11	19.20	9.50	1.48
β-casein (%) #	68.97	34.13	54.80	65.00	78.96
κ-casein (%) #	17.24	12.30	20.40	3.50	1.78

^a^: Adapted from Miranda et al. (2004) [21]; ^b^: adapted from Lönnerdal et al. (2003) [22]; ^c^: adapted from Uniacke-Lowe et al. (2010) [6]; ^d^: adapted from Selvaggi et al. (2014) [8]; ^e^: adapted from Brezovečki et al. (2015) [9]; “*”: the casein content of the total protein; and “#”: the α_S1_-CN, α_S2_-CN, and κ-CN content of the casein, respectively. There are no reports on the presence of α_S2_-CN in HM [6,21,22].

**Table 2:** Predicting results of the physicochemical properties of allergenic subtypes (α_S1_-CN, α_S2_-CN, and κ-CN), respectively, in CM, GM, CAM, and MM, as well as their accession numbers in the UniProtKB database.

Species Name	Protein	UniProtKB Database Accession Number	Molecular Weight/Da (Mw)	Grand Average of Hydropathicity (GRAVY)	Aliphatic Index(AI)
Cow	α_s1_-casein	P02662	24,528.94	−0.481	85.19
Goat		P18626	24,289.59	−0.534	80.23
Camel		O97943	26,861.40	−0.661	84.30
Mare		Q95KZ7	24,688.89	−0.801	80.67
Cow	α_s2_-casein	P02663	26,018.69	−0.704	73.74
Goat		P33049	26,389.03	−0.844	66.46
Camel		O97944	22,964.10	−0.661	67.62
Mare		A0A0C5DH76	27,262.89	−0.729	70.00
Cow	κ-casein	P02668	21,269.35	−0.287	81.63
Goat		P02670	21,441.32	−0.328	79.27
Camel		P79139	20,417.56	−0.150	90.49
Mare		P82187	21,021.43	−0.191	97.41

Note: GRAVY evaluates the hydrophilicity and hydrophobicity of proteins, where negative and positive values of GRAVY indicate good hydrophilicity or hydrophobicity, respectively [27]. In addition, AI evaluates the thermal stability of proteins [28], wherein the AI value that is greater than 65 indicates good thermal stability [29]. Lastly, both GRAVY and AI utilize units of 1.

**Table 3:** The number and proportion between the hydrolyzed peptides and the allergenic peptides from allergenic subtypes in CM, GM, CAM, and MM, respectively. Among them, the hydrolyzed peptides and allergenic peptides were generated by simulating gastrointestinal (GI) tract hydrolysis, as well as allergenic peptide prediction, respectively. Allergenic peptides obtained from the linear B-cell epitope amino acid sequence were considered allergenic peptides that were derived from the linear B-cell epitope.

Species Name	Protein	Hydrolyzed Peptide Number	Allergenic Peptide Number		Allergenic Peptide Number/Hydrolyzed Peptide Number (%)
			Total	Liner B-Cell Epitope	
Cow	*α_s1_*-casein	30	12	8	40.00
	*α_s2_*-casein	42	19	6	45.24
	*κ*-casein	24	11	6	45.83
Goat	*α_s1_*-casein	31	11	6	35.48
	*α_s2_*-casein	42	15	6	35.71
	*κ*-casein	23	9	3	39.13
Camel	*α_s1_*-casein	36	11	4	30.56
	*α_s2_*-casein	32	13	7	40.62
	*κ*-casein	22	3	1	13.63
Mare	*α_s1_*-casein	35	9	2	25.71
	*α_s2_*-casein	38	11	5	28.95
	*κ*-casein	21	5	3	23.81

**Table 4:** The predicted results of the linear T cell epitopes in regard to the allergenic subtypes, respectively, in CM, GM, CAM, and MM.

Protein Type	Species	Consensus Core Epitope	Binding Type	T-Cell Epitope Number
α_s1_-casein	Cow	IGSESTEDQ, SESTEDQAM	Strong binder, Weak binder	12
	Goat	IGSESTEDQ, SESTEDQAM		5
	Camel	-		4
	Mare	-		6
α_s2_-casein	Cow	MEHVSSSEE, VRNANEEEY, EYSIGSSSE, IGSSSEESA	Weak binder	12
	Goat	MEHVSSSEE, VRNANEEEY, EYSIGSSSE, IGSSSEESA	Weak binder	10
	Camel	-	-	9
	Mare	-	-	6
κ-casein	Cow	FLGAEVQNQ, PYYAKPAAV	Weak binder	13
	Goat	FLGAEVQNQ, PYYAKPAAV	Weak binder	13
	Camel	FLGAEVQNQ, INTVATVEP	Weak binder	11
	Mare	FLGAEVQNQ, INTVATVEP	Weak binder	12

## Data Availability

The data that support the findings of this study are available from the corresponding author upon reasonable request.

## Supplementary Materials

The following supporting information can be downloaded at: https://www.mdpi.com/article/10.3390/ijms24032481/s1.

## Abbreviations

CM	cow milk
GM	goat milk
CAM	camel milk
MM	mare milk
HM	human milk
α_S1_-CN	α_S1_-casein
α_S2_-CN	α_S2_-casein
β-CN	β-casein
κ-CN	κ-casein
α-CN	α-casein
β-lg	β-lactoglobulin
GI	gastrointestinal
GRAVY	grand average of hydropathicity
AI	aliphatic index
NRPFLB-cellE	number of recognized polypeptides fragments of linear B-cell epitope
