# Supplementary material for: Secrets behind Protein Sequences: Unveiling the Potential Reasons for Varying Allergenicity Caused by Caseins from Cows, Goats, Camels, and Mares Based on Bioinformatics Analyses
Source: Int J Mol Sci. 2023 Jan 27;24(3):2481. doi: 10.3390/ijms24032481 (PMC9916876; doi:10.3390/ijms24032481)
Supplement: Supplementary file 1 [file ijms-24-02481-s001.zip › ijms-2119626-supplementary.pdf]

Table S1. Predicting results of the physicochemical properties of allergic subtypes (α_{S1}-CN, α_{S2}-CN and κ-CN), respectively, in CM, GM, CAM and MM.

Scientific name		α_{S1} -casein			
		Cow	Goat	Camel	Mare
Number of amino acids		214	214	230	208
Formula		$C_{1108}H_{1719}N_{281}O_{333}S_7$	$C_{1092}H_{1694}N_{284}O_{329}S_7$	$C_{1207}H_{1873}N_{315}O_{367}S_6$	$C_{1102}H_{1719}N_{301}O_{334}S_5$
Molecular weight/Da		24528.94	24289.59	26861.40	24688.89
Theoretical PI		4.98	5.32	4.96	5.57
Total number of negatively charged residues (Asp + Glu)		32	27	38	33
Total number of positively charged residues (Arg + Lys)		21	21	25	26
Grand average of hydropathicity (GRAVY)		-0.481	-0.534	-0.661	-0.801
Aliphatic index		85.19	80.23	84.3	80.67
Amino acid composition	Ala(A)	5.6%	7.0%	4.3%	4.3%
	Arg(R)	2.8%	3.3%	4.8%	4.8%
	Asn(N)	3.7%	5.1%	3.0%	3.0%
	Asp(D)	3.3%	3.3%	5.2%	5.2%
	Cys(C)	0.5%	0.5%	0.4%	0.4%
	Gln(Q)	6.5%	6.5%	7.0%	7.0%
	Glu(E)	11.7%	9.3%	11.3%	11.3%
	Gly(G)	4.2%	4.2%	2.6%	2.6%
	His(H)	2.3%	1.9%	2.6%	2.6%
	Ile(I)	5.6%	4.7%	5.2%	5.2%
	Leu(L)	10.3%	10.3%	10.4%	10.4%
	Lys(K)	7.0%	6.5%	6.1%	6.1%

Met(M)	2.8%	2.8%	2.2%	2.2%
Phe(F)	3.7%	3.3%	2.6%	2.6%
Pro (P)	7.9%	8.9%	8.3%	8.3%
Ser (S)	7.5%	8.4%	7.0%	7.0%
Thr (T)	2.8%	2.8%	3.9%	3.9%
Trp (W)	0.9%	0.9%	1.3%	1.3%
Tyr(Y)	4.7%	5.1%	5.2%	5.2%
Val(V)	6.1%	5.1%	6.5%	6.5%

Table S1. Continued

Scientific name		α_{S2}-casein			
		Cow	Goat	Camel	Mare
Number of amino acids		214	214	230	208
Formula		$C_{1108}H_{1719}N_{281}O_{333}S_7$	$C_{1092}H_{1694}N_{284}O_{329}S_7$	$C_{1207}H_{1873}N_{315}O_{367}S_6$	$C_{1102}H_{1719}N_{301}O_{334}S_5$
Molecular weight/Da		24528.94	24289.59	26861.40	24688.89
Theoretical PI		4.98	5.32	4.96	5.57
Total number of negatively charged residues (Asp + Glu)		32	27	38	33
Total number of positively charged residues (Arg + Lys)		21	21	25	26
Grand average of hydropathicity (GRAVY)		-0.481	-0.534	-0.661	-0.801
Aliphatic index		85.19	80.23	84.3	80.67
Amino acid composition	Ala(A)	5.6%	7.0%	4.3%	4.3%
	Arg(R)	2.8%	3.3%	4.8%	4.8%
	Asn(N)	3.7%	5.1%	3.0%	3.0%
	Asp(D)	3.3%	3.3%	5.2%	5.2%
	Cys(C)	0.5%	0.5%	0.4%	0.4%
	Gln(Q)	6.5%	6.5%	7.0%	7.0%
	Glu(E)	11.7%	9.3%	11.3%	11.3%
	Gly(G)	4.2%	4.2%	2.6%	2.6%
	His(H)	2.3%	1.9%	2.6%	2.6%
	Ile(I)	5.6%	4.7%	5.2%	5.2%
	Leu(L)	10.3%	10.3%	10.4%	10.4%
	Lys(K)	7.0%	6.5%	6.1%	6.1%
	Met(M)	2.8%	2.8%	2.2%	2.2%
	Phe(F)	3.7%	3.3%	2.6%	2.6%

	Pro(P)	7.9%	8.9%	8.3%	8.3%
	Ser(S)	7.5%	8.4%	7.0%	7.0%
	Thr(T)	2.8%	2.8%	3.9%	3.9%
	Trp(W)	0.9%	0.9%	1.3%	1.3%
	Tyr(Y)	4.7%	5.1%	5.2%	5.2%
	Val(V)	6.1%	5.1%	6.5%	6.5%

Table S1. Continued

Scientific name		κ-casein			
		Cow	Goat	Camel	Mare
Number of amino acids		190	192	182	185
Formula		$C_{962}H_{1503}N_{245}O_{286}S_6$	$C_{963}H_{1495}N_{249}O_{293}S_6$	$C_{933}H_{1470}N_{240}O_{267}S_3$	$C_{974}H_{1511}N_{245}O_{265}S_4$
Molecular weight/Da		21269.35	21441.32	20417.56	21021.43
Theoretical PI		6.29	5.53	8.55	8.51
Total number of negatively charged residues (Asp + Glu)		16	18	14	12
Total number of positively charged residues (Arg + Lys)		15	14	16	14
Grand average of hydropathicity (GRAVY)		-0.287	-0.328	-0.15	-0.091
Aliphatic index		81.63	79.27	90.49	97.41
Amino acid composition	Ala(A)	8.4%	9.4%	7.7%	5.9%
	Arg(R)	2.6%	2.6%	4.4%	3.2%
	Asn(N)	4.2%	4.7%	5.5%	5.4%
	Asp(D)	2.1%	3.6%	1.1%	1.6%
	Cys(C)	1.1%	1.6%	1.1%	1.1%
	Gln(Q)	7.9%	7.8%	6.6%	5.9%
	Glu(E)	6.3%	5.7%	6.6%	4.9%
	Gly(G)	1.6%	1.0%	1.1%	0.5%
	His(H)	1.6%	2.1%	1.1%	3.2%
	Ile(I)	6.8%	5.7%	8.8%	9.2%
	Leu(L)	6.8%	6.8%	5.5%	7.0%
	Lys(K)	5.3%	4.7%	4.4%	4.3%
	Met(M)	2.1%	1.6%	0.5%	1.1%

Phe(F)	3.7%	3.6%	4.9%	3.8%
Pro(P)	11.1%	10.4%	12.6%	14.1%
Ser(S)	7.4%	7.3%	4.4%	4.9%
Thr(T)	8.9%	8.9%	10.4%	8.1%
Trp(W)	0.5%	0.5%	0.0%	0.5%
Tyr(Y)	4.7%	4.7%	3.8%	5.4%
Val(V)	6.8%	7.3%	9.3%	9.7%

Table S2. Predicting results of linear B-cell epitope prediction based on two different databases

Protein type	Species	Prediction server	Predicting regional/aa
α $_{s1}$-casein	Cow	BepiPred1.0 Server	18-26,28-29,45-50,52-54,56-70,74-75,77-104,126-133,143-152,173-179,185-210
		IEDB Server	18-26,28-29,45-50,52-54,56-70,74-75,77-104,126-133,142-152,173-179,185-210
	Goat	BepiPred1.0 Server	18-33,57-104,126-134,136-148,173-177,186-210
		IEDB Server	18-33,57-104,126-134,136-148,173-177,186-210
	Camel	BepiPred1.0 Server	18-22,26-35,59-110,154-168,194-195,205-226
		IEDB Server	18-22,26-35,59-110,154-168,194-195,205-226
	Mare	BepiPred1.0 Server	21-22,24-36,63-109,158-163,186-188,196-203
		IEDB Server	21-22,24-36,63-109,158-163,186-188,196-205
α $_{s2}$-casein	Cow	BepiPred1.0 Server	20-30,33-38,40-49,60-81,86-87,91-93,125-129,131-157,161-162,167-173,206-214
		IEDB Server	20-30,33-38,40-49,60-81,86-87,91-93,125-129,131-157,161-162,167-173,206-214
	Goat	BepiPred1.0 Server	22-29,60-84,86-87,92-94,113-117,119-120,126-158,162-163,168-174,201-217
		IEDB Server	22-29,61-84,86-87,92-94,113-117,119-120,126-158,162-163,168-174,201-217
	Camel	BepiPred1.0 Server	19-31,44-50,65-77,103-110,116-119,122-141,144-153,181-184
		IEDB Server	19-31,44-50,65-77,102-110,116-119,122-141,144-153,181-184
	Mare	BepiPred1.0 Server	20-31,34-37,46-51,56-66,68-98,135-145,159-168,170-184,208-220
		IEDB Server	20-31,34-37,46-51,56-66,68-98,135-145,159-168,170-184,208-220
	Cow	BepiPred1.0 Server	21-33,81-90,103-121,130-141,145-162,165-183
		IEDB Server	21-33,81-90,103-121,130-141,145-162,165-183
	Goat	BepiPred1.0 Server	21-32,37-38,81-94,102-119,130-142,146-192
		IEDB Server	21-32,37-38,81-94,102-119,130-142,146-192

κ -casein	Camel	BepiPred1.0 Server	22-30,55-58,76-83,98-135,137-152,163-177
		IEDB Server	22-30,55-58,76-83,98-135,137-152,163-177
	Mare	BepiPred1.0 Server	22-33,55-60,80-88,108-115,119-120,123-125,138-160,169-180
		IEDB Server	22-33,55-60,80-88,108-115,119-120,123-125,138-160,169-180

Table S3. Identical or similar linear B-cell epitope fragments in allergenic subtypes of four different species milks

NO.	linear B-cell epitope fragments	Source
1	KHPIKHQGL	α_{s1}-CN for CM
	KHPINHRGLSPEVPNE	α_{s1}-CN for GM
2	AYPSGAW	α_{s1}-CN for CM
	AYPSG	α_{s1}-CN for GM
3	KHY	α_{s2}-CN for CM and GM
4	DQVKR	α_{s2}-CN for CM
	DQVKRNAGPFTPTVNREQLSTS EENSKKTIDME	α_{s2}-CN for GM
5	VF	α_{s2}-CN for CM and GM
6	KLTEEEK	α_{s2}-CN for CM and GM
7	VQNQEQPTC	κ-CN for CAM
	VQNQEQPTCHKN	κ-CN for MM

Table S4. Predicting results of secondary structure in allergenic subtypes of CM, GM, CAM and MM.

Scientific name	Protein	α-helix	β-sheet	β-turn	random coil
Cow	α_{s1}-casein	44.39%	4.67%	1.40%	49.53%
Goat		42.06%	7.01%	3.27%	47.66%
Camel		50.43%	7.39%	3.04%	39.13%
Mare		48.08%	5.77%	3.85%	42.31%
Cow	α_{s2}-casein	48.20%	4.95%	2.25%	44.59%
Goat		47.53%	7.17%	3.14%	42.15%
Camel		60.10%	5.18%	2.07%	32.64%
Mare		45.02%	6.49%	0.87%	47.62%
Cow	κ-casein	15.26%	10.00%	1.05%	73.68%
Goat		19.27%	10.94%	1.04%	68.75%
Camel		13.74%	15.38%	1.10%	69.78%
Mare		13.51%	15.14%	1.08%	70.27%

Table S5. Source, sequence, physicochemical properties, content and solubility of allergenic peptides. The allergenic peptides produced by after simulation GI hydrolysis produced of allergenic subtypes of CM, GM, CAM and MM.

Protein Species	Species name	Position of cleavage site	Peptide sequence	Peptide mass (Da)	Length	Content(%)	Estimated solubility
		0,2	MK	277.38	2	3.33	Good
		3,5	ARPK	470.57	4	3.33	Good
		14,18	HPIK	493.61	4	3.33	Good
		47,49	GK	203.24	2	3.33	Good
		49,51	SK	233.27	2	3.33	Good
	Cow	79,94	QMEAESISSSEEIVPN SVEQK	2321.50	21	3.33	Good
		124,134	EIVPNSAEER	1143.22	10	3.33	Good
		135,139	HSMK	501.60	4	3.33	Good
		139,147	EGIHAQQK	910.00	8	3.33	Good
		147,156	EPMIGVNQE	1016.13	9	3.33	Good
		161,164	PEL	357.41	3	3.33	Good
		188,194	TDAPSF	636.66	6	3.33	Good
		0,2	MK	277.38	2	3.23	Good
		14,18	ARPK	470.57	4	3.23	Good
		24,34	GLSPEVPNEN	1055.11	10	3.23	Good
		55,57	SK	233.27	2	3.23	Good
		73,76	QMK	405.51	3	3.23	Good
	Goat	76,94	AGSSSSSEEIVP NSAEQK	1806.86	18	3.23	Good
		124,128	NVPQL	569.658	5	3.23	Good
		129,134	SAEEQ	562.53	5	3.23	Good
		135,139	HSMK	501.60	4	3.23	Good
		147,156	QPMIAVNQE	1029.18	9	3.23	Good
		188,194	TDAPSF	636.66	6	3.23	Good

α_{s1}-casein	Camel	0,2	MK	277.38	2	2.78	Good
		14,18	ARPK	470.57	4	2.78	Good
		27,37	QNEPDSIEEV	1159.17	10	2.78	Good
		38,40	NK	260.29	2	2.78	Good
		111,114	LE1E	389.41	3	2.78	Good
		115,117	HR	311.34	2	2.78	Good
		130,133	DQK	389.41	3	2.78	Good
		137,139	VK	245.32	2	2.78	Good
		151,160	INEDNHPQL	1079.13	9	2.78	Good
		185,189	GASP	330.34	4	2.78	Good
		209,222	DTPEGIASEDGGK	1275.29	13	2.78	Good
	Mare	0,2	MK	277.38	2	2.86	Good
		34,36	EK	275.31	2	5.71	Good
		54,56	NR	288.31	2	2.86	Good
		62,64	EK	275.31	2	5.71	Good
		107,110	IPR	384.48	3	2.86	Good
		110,113	EDM	393.41	3	2.86	Good
		128,130	NQ	260.25	2	2.86	Good
		144,151	VVNQEQA	786.84	7	2.86	Good
		164,167	DVY	395.41	3	2.86	Good
	Cow	0,2	MK	277.38	2	2.38	Good
		16,34	NTMEHVSSSEESIISQ ET	2008.10	18	2.38	Good
		39,47	NMAINPSK	874.02	8	2.38	Good
		47,50	ENL	374.39	3	2.38	Good
		54,56	CK	249.33	2	2.38	Good
		56,60	EVVR	501.58	4	2.38	Good
		96,102	LNEINQ	729.79	6	2.38	Good
		124,128	DQVK	488.54	4	2.38	Good
		138,140	NR	288.31	2	4.76	Good

		140,143	EQL	388.42	3	2.38	Good
		152,161	TVDMESTEV	1010.08	9	2.38	Good
		162,164	TK	247.29	2	7.14	Good
		165,167	TK	247.29	2	7.14	Good
		173,175	NR	288.31	2	4.76	Good
		189,191	AL	202.25	2	2.38	Good
		199,203	QHQK	539.59	4	2.38	Good
		208,212	IQPK	484.60	4	2.38	Good
		212,214	TK	247.29	2	7.14	Good
		218,220	VR	273.34	2	2.38	Good
		0,2	MK	277.38	2	2.38	Good
		16,18	HK	283.33	2	2.38	Good
		48,50	EK	275.31	2	2.38	Good
		51,61	CTTSCEEVVR	1126.27	10	2.38	Good
		68,71	SIR	374.44	3	2.38	Good
		97,103	LNEINQ	729.79	6	2.38	Good
		125,129	DQVK	488.54	4	2.38	Good
α_{s2}-casein	Goat	135,141	TPTVNR	686.77	6	2.38	Good
		141,144	EQL	388.42	3	2.38	Good
		153,162	TIDMESTEV	1024.11	9	2.38	Good
		163,165	TK	247.29	2	4.76	Good
		166,168	TK	288.31	2	4.76	Good
		174,176	NR	288.31	2	2.38	Good
		197,204	TVDQHQK	854.92	7	2.38	Good
		219,221	VR	273.34	2	2.38	Good
		0,2	MK	277.38	2	3.13	Good
	Camel	16,35	HEMDQGSSSEE SINVSQQK	2120.19	19	3.13	Good
		37,40	QVK	373.45	3	3.13	Good
		41,48	VAIHPSK	750.90	7	3.13	Good

Mare	48,54	EDICST	666.70	6	3.13	Good
	64,76	EVESAEVPTENK	1331.40	12	3.13	Good
	90,92	QA	217.23	2	3.13	Good
	113,121	IPTVNTEQ	900.98	8	3.13	Good
	122,139	SISEESTEVPTEES TEV	1852.88	17	3.13	Good
	140,142	TK	247.29	2	3.13	Good
	151,155	DHQK	526.55	4	3.13	Good
	164,166	QT	247.25	2	3.13	Good
	169,172	PEY	407.42	3	3.13	Good
	0,2	MK	277.38	2	2.63	Good
	88,92	PTER	501.54	4	2.63	Good
	95,100	EVEEK	632.67	5	2.63	Good
	113,115	EK	275.31	2	5.26	Good
	122,124	QA	217.23	2	2.63	Good
	135,139	DQTK	490.51	4	2.63	Good
	163,176	TVDMESTEVVTE K	1467.61	13	2.63	Good
	192,194	EK	275.31	2	5.26	Good
	205,214	QHQTTMDPR	1113.21	9	2.63	Good
	214,217	SHR	398.42	3	2.63	Good
	218,221	TNS	320.30	3	2.63	Good
Cow	0,3	MMK	408.58	3	4.17	Good
	19,31	GAQEQNQEQPIR	1397.47	12	4.17	Good
	31,34	CEK	378.44	3	4.17	Good
	34,37	DER	418.41	3	4.17	Good
	42,45	IAK	330.43	3	4.17	Good
	53,55	SR	264.28	2	4.17	Good
	64,70	QQKPVA	669.78	6	4.17	Good
	82,89	AKPAAVR	711.86	7	4.17	Good

		107,118	SCQAQPTTMAR	1193.36	11	4.17	Good
		133,137	NQDK	503.51	4	4.17	Good
		137,166	TEIPTINTIASGEPTS TPTTEAVESTVAT	2919.14	29	4.17	Good
κ-casein	Goat	0,3	MMK	408.58	3	4.35	Good
		19,34	GAQEQNQEQPICCE K	1704.85	15	4.35	Good
		34,37	DER	418.41	3	4.35	Good
		42,45	IAK	330.43	3	4.35	Good
		53,55	SR	261.28	2	4.35	Good
		64,70	QQRPVA	697.79	6	4.35	Good
		107,115	SCQDQPTT	878.91	8	4.35	Good
		116,118	AR	245.28	2	4.35	Good
		133,137	NQDK	504.50	4	4.35	Good
	Camel	0,2	MK	277.38	2	4.55	Good
		31,33	EK	275.31	2	4.55	Good
		81,88	AKPVAIR	753.94	7	4.55	Good
	Mare	0,2	MK	277.38	2	4.76	Good
		18,32	GAEVQNQEQPTCHK	1568.68	14	4.76	Good
		52,57	NSSPR	559.58	5	4.76	Good
		58,62	EPIY	520.58	4	4.76	Good
		103,109	PSTVVR	657.77	6	4.76	Good

Table S6. Identical allergenic peptide fragments in allergenic subtypes of four different species milks.

NO.	Allergenic peptides	Source
1	MK	α_{s1}-CN for CM, GM, CAM and MM<br>α_{s2}-CN for CM, GM, CAM and MM<br>κ-CN for CAM and MM
2	MMK	κ-CN for CM and GM
3	ARPK	α_{s1}-CN for CM, GM and CAM
4	EK	α_{s1}-CN for MM<br>α_{s2}-CN for GM and MM<br>κ-CN for CAM
5	NR	α_{s1}-CN for MM<br>α_{s2}-CN for CM and GM
6	SK	α_{s1}-CN for CM and GM
7	IAK	κ-CN for CM and GM
8	QA	α_{s2}-CN for CAM and MM
9	SR	κ-CN for CM and GM
10	LNEINQ	α_{s2}-CN for CM and GM
11	DQVK	α_{s2}-CN for CM and GM
12	HSMK	α_{s1}-CN for CM and GM
13	EQL	α_{s2}-CN for CM and GM
14	NQDK	κ-CN for CM and GM
15	TK	α_{s2}-CN for CM, GM and CAM
16	VR	α_{s2}-CN for CM and GM

Table S7. Predicting results of the linear T cell epitopes of allergenic subtypes in CM, GM, CAM and MM.

Protein type	Species	Core Epitope	Binding Type	Linear T cell epitope number
αs1-casein	Cow	IGSESTEDQ	Strong binder	12
		SESTEDQAM	Weak binder	
		IKQMEAESI	Weak binder	
		AESISSSEE	Weak binder	
		SISSSEEIV	Weak binder	
		LEIVPNSAE	Weak binder	
		MIGVNQELA	Weak binder	
		IGVNQELAY	Weak binder	
		LAYFYPELF	Weak binder	
		WYYVPLGTQ	Weak binder	
		YVPLGTQYT	Weak binder	
		YTDAPSFSD	Weak binder	
	Goat	IGSESTEDQ	Strong binder	5
		SESTEDQAM	Weak binder	
		IAVNQELAY	Weak binder	
		LAYFYPQLF	Weak binder	
		YTDAPSFSD	Weak binder	
	Camel	PDSIEEVLN	Weak binder	4
		VSSTTEQKD	Weak binder	
		VMQYIAHPS	Weak binder	
		PEGIASEDG	Weak binder	
	Mare	PEIIQNEQD	Weak binder	6
		IQNEQDSRE	Weak binder	
		LEYINELNR	Weak binder	
		VVPINTEKR	Strong binder	
		INTEKRIPR	Weak binder	
		MLYQHTLEQ	Weak binder	
	Cow	MEHVSSSEE	Weak binder	12
		EESIISQET	Weak binder	
		IISQETYKQ	Strong binder	
		VRNANEEEY	Weak binder	
		EYSIGSSSE	Weak binder	
		IGSSSEESA	Weak binder	
		EESAEVATE	Strong binder	
		SAEVATEEV	Weak binder	
		VATEEVKIT	Weak binder	
		TEEVKITVD	Weak binder	
		VKITVDDKH	Weak binder	
		ITVDDKHYQ	Weak binder	
	Goat	MEHVSSSEE	Weak binder	10
		VRNANEEEY	Weak binder	

		EYSIRSSSE	Weak binder	
		IRSSSEESA	Weak binder	
		EESAEVAPE	Strong binder	
		PEEIKITVD	Weak binder	
		IKITVDDKH	Weak binder	
		ITVDDKHYQ	Weak binder	
		QLSTSEENS	Weak binder	
		IDMESTEVF	Weak binder	
		EESINVSQQ	Weak binder	
		VKKVAIHPS	Weak binder	
α_{s2}-casein		VRNIKEVES	Weak binder	
		IKEVESAEV	Weak binder	
	Camel	VESAEVPTE	Strong binder	9
		VNTEQLSIS	Weak binder	
		QLSISEEST	Weak binder	
		EESTEVPTE	Weak binder	
		TELTEEEKD	Weak binder	
		EDSVNISQE	Weak binder	
		YVVIPTSKE	Strong binder	
		VYSSSSSSE	Weak binder	
	Mare	MESTEVVTE	Weak binder	6
		VVTEKTELT	Weak binder	
		EKTELTEEE	Weak binder	
		FLGAQEQNQ	Weak binder	
		EQPIRCEKD	Weak binder	
		IRCEKDERF	Weak binder	
		FFSDKIAKY	Weak binder	
		VALINNQFL	Weak binder	
		PYYAKPAAV	Weak binder	
	Cow	IPTINTIAS	Weak binder	13
		PTTEAVEST	Weak binder	
		VESTVATLE	Strong binder	
		ESTVATLED	Strong binder	
		VATLEDSPE	Strong binder	
		PEVIESPPE	Weak binder	
		INTVQVTST	Weak binder	
		FLGAQEQNQ	Weak binder	
κ-casein		PYYAKPVAV	Weak binder	
		AINTIASAE	Weak binder	
		INTIASAEP	Weak binder	
	Goat	IASAEPTVH	Weak binder	13
		VHSTPTTEA	Weak binder	
		PTTEAIVNT	Weak binder	
		TEAIVNTVD	Weak binder	
		IVNTVDNPE	Strong binder	
		VNTVDNPEA	Weak binder	

	EASSESIAS	Weak binder	
	SESIASASE	Weak binder	
	IASASETNT	Weak binder	
Camel	FLGAEVQNQ	Strong binder	11
	VKYFPIQFV	Weak binder	
	PNYAKPVAI	Weak binder	
	PAINTVATV	Weak binder	
	INTVATVEP	Weak binder	
	VATVEPPVI	Strong binder	
	VNTVVIAEA	Weak binder	
	VVIAEASSE	Weak binder	
	IAEASSEFI	Weak binder	
	ITTSTPETT	Weak binder	
	PETTTVQIT	Weak binder	
Mare	FLGAEVQNQ	Strong binder	12
	YYVLNSSPR	Weak binder	
	QYYARPAAV	Weak binder	
	PAAVRPHVQ	Weak binder	
	INTIATVEP	Weak binder	
	IATVEPTPI	Strong binder	
	TVNNAVIPD	Weak binder	
	VNNAVIPDA	Weak binder	
	NNAVIPDAS	Weak binder	
	VIPDASSEF	Weak binder	
	FIIASTPET	Weak binder	
	PETTTVPVT	Weak binder	

Figure S1. Alignments of amino acid sequences of caseins derived from different species milks. The αS1-CN and κ-CN of HM were used as template, where panels A and B show aligned amino acid sequence of αS1-CN and κ-CN, respectively. The height of the bar plots under each sequence alignment represents the number of identical amino acids from four different species milks (Maximum four, minimum zero). The number marked on the left was the UniprotKB database accession number.

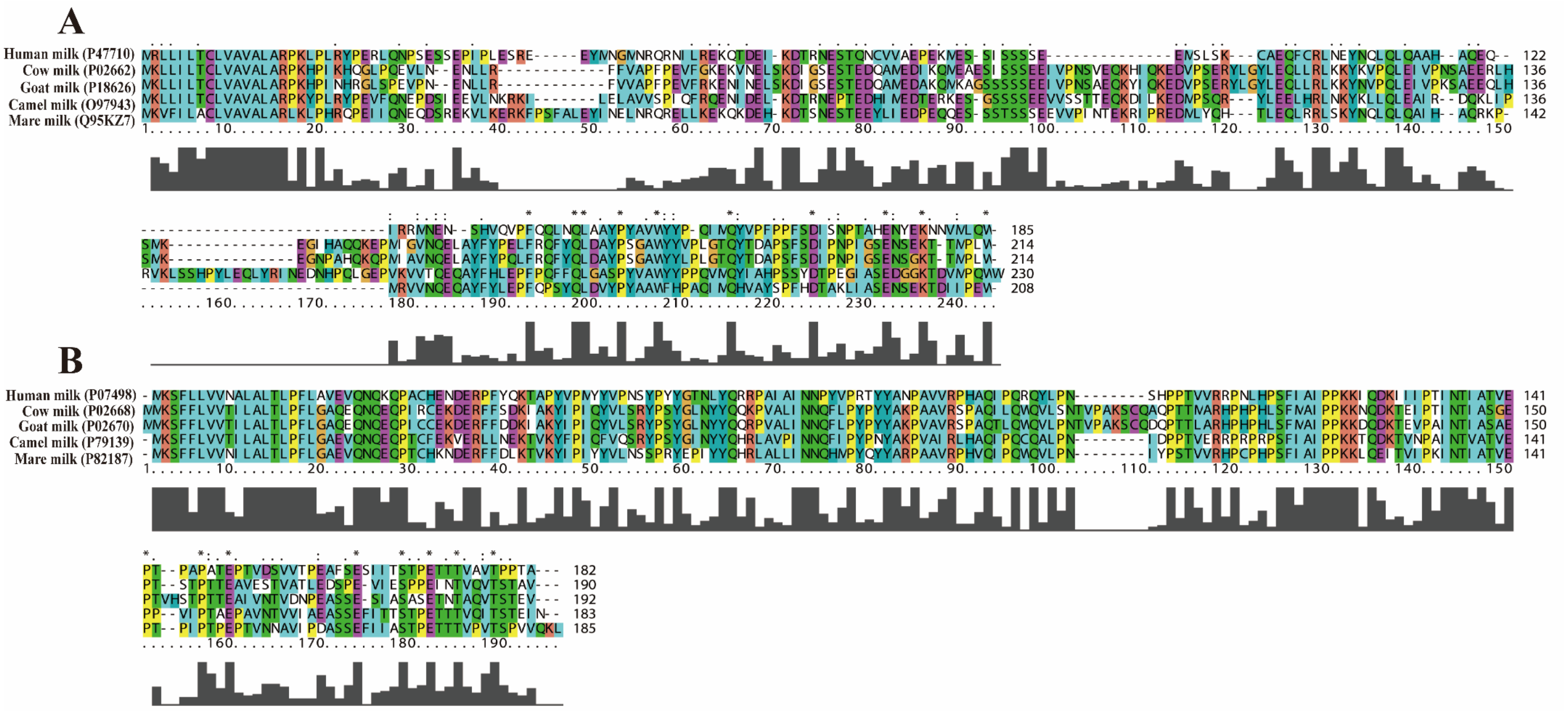

Figure S2. Predicting results of polarity and distribution of amino acids in allergenic subtypes of CM, GM, CAM and MM. Panels A to D represent CM, GM, CAM and MM, respectively.

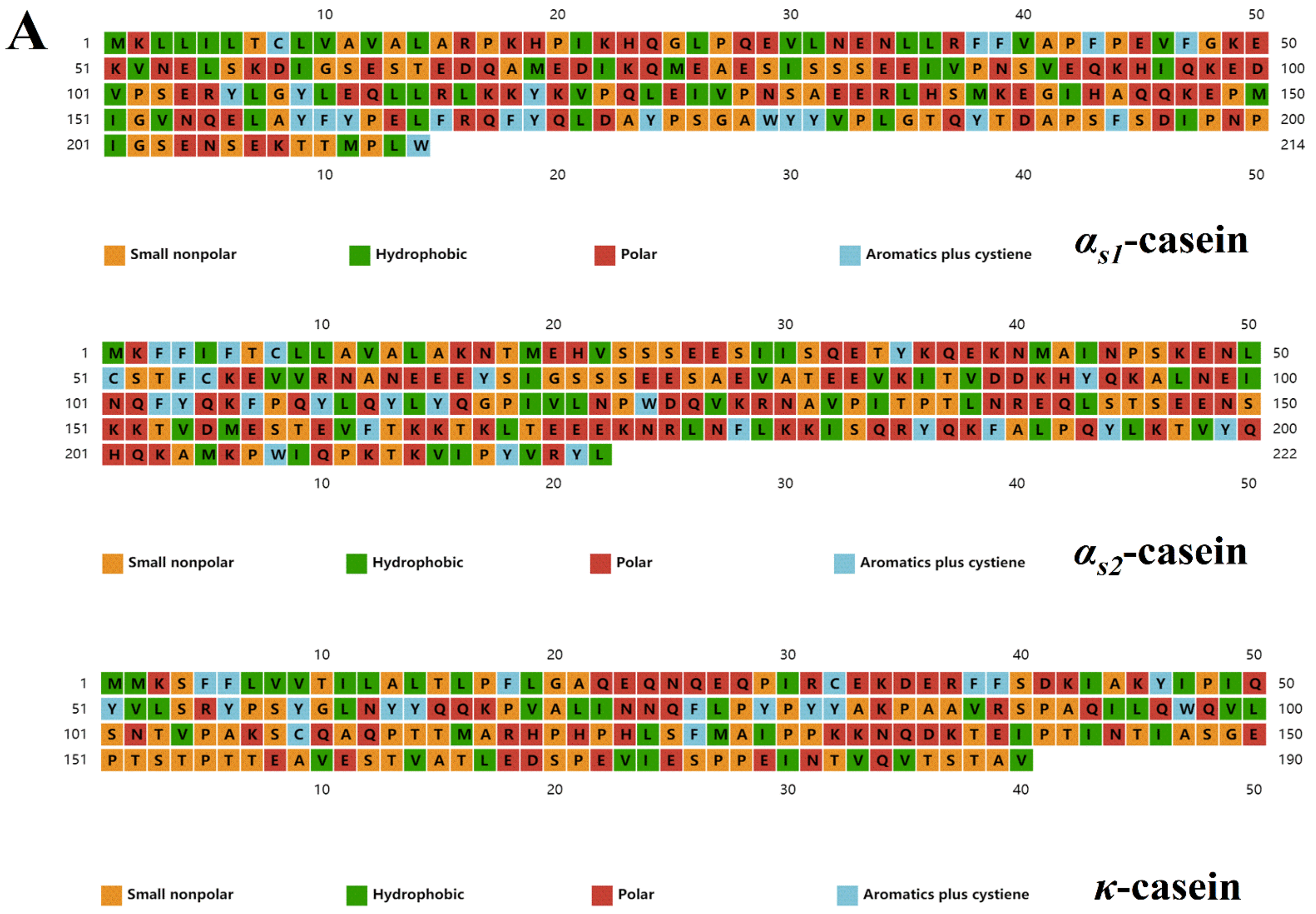

Figure S2. Continued

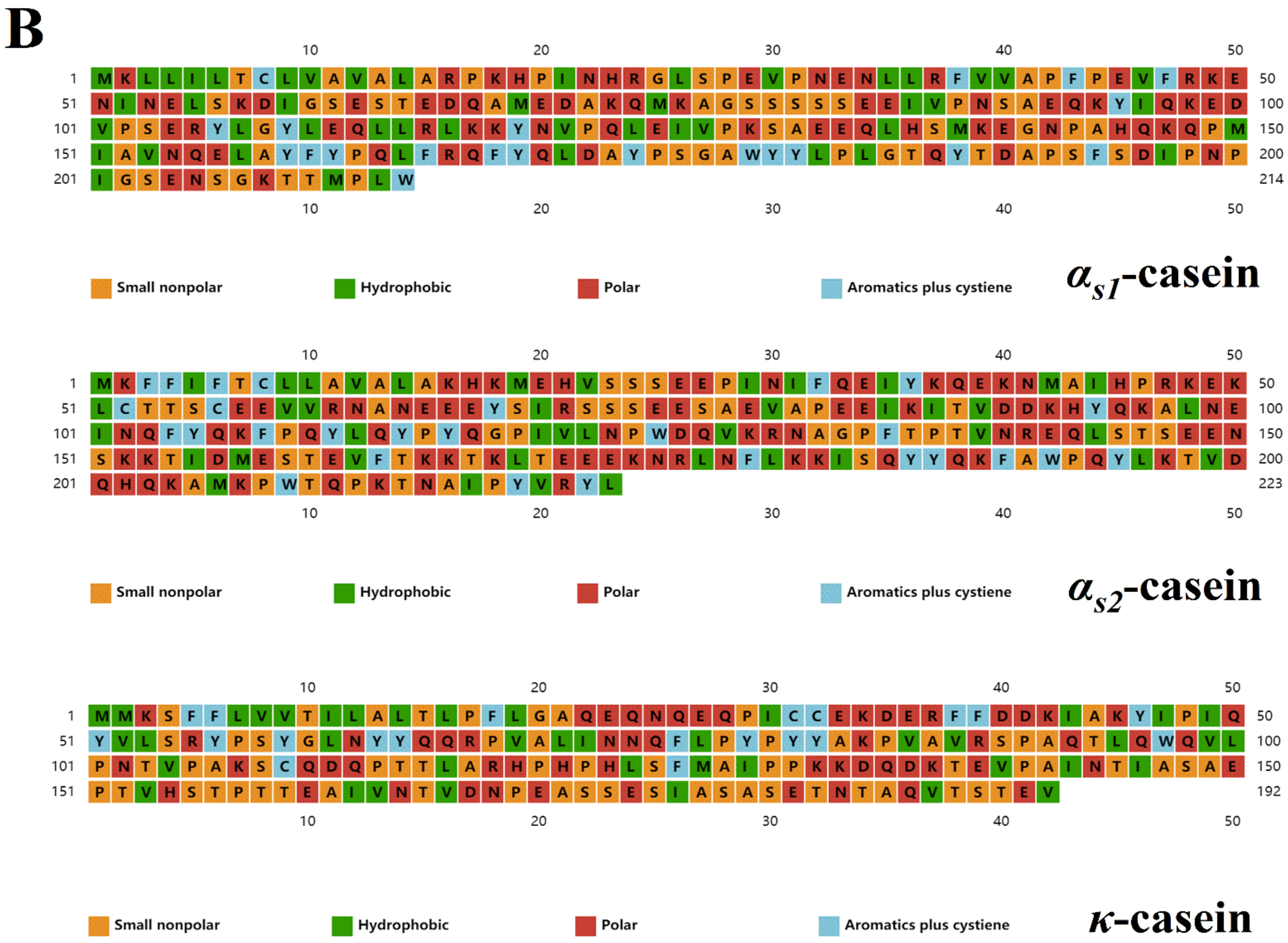

Figure S2. Continued

C

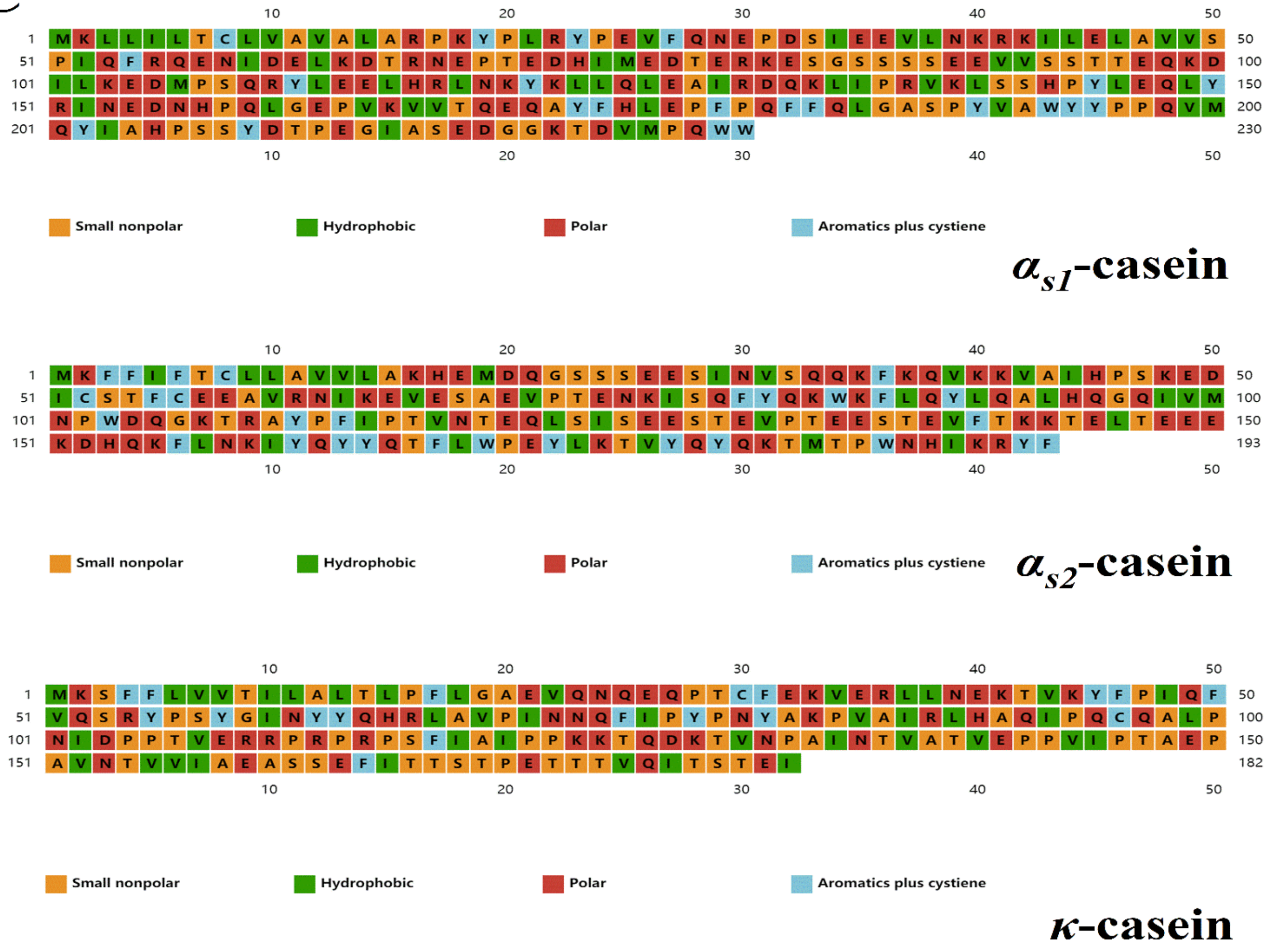

Figure S2. Continued

D

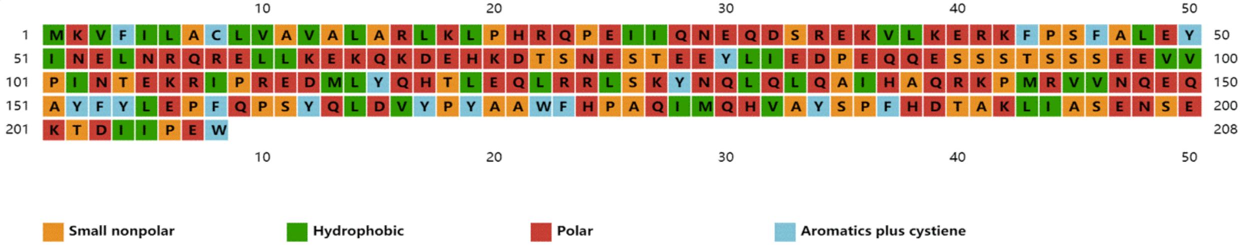

α_{s1}-casein

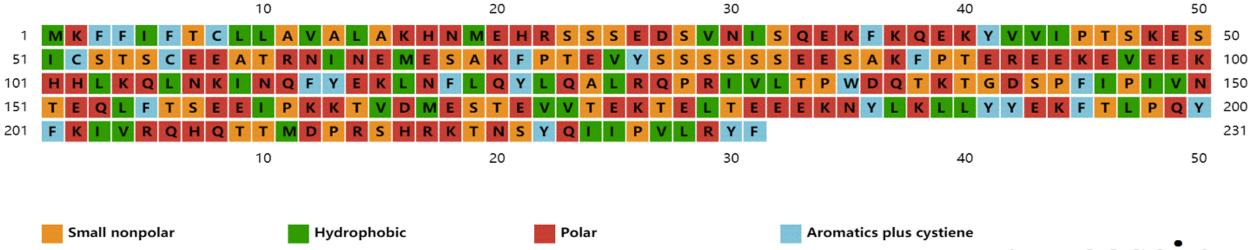

α_{s2}-casein

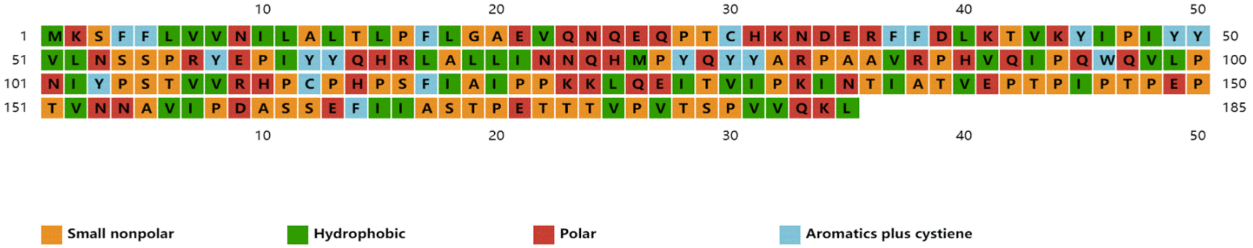

κ-casein

Figure S3. Predicting results of regional and sequence fragments of linear B-cell epitope in allergenic subtypes sequences of CM, GM, CAM and MM. Panels A to D represent CM, GM, CAM and MM, respectively. Among them, linear B-cell epitopes red location marked in CM are considered cross-sequences with reported corresponding epitopes.

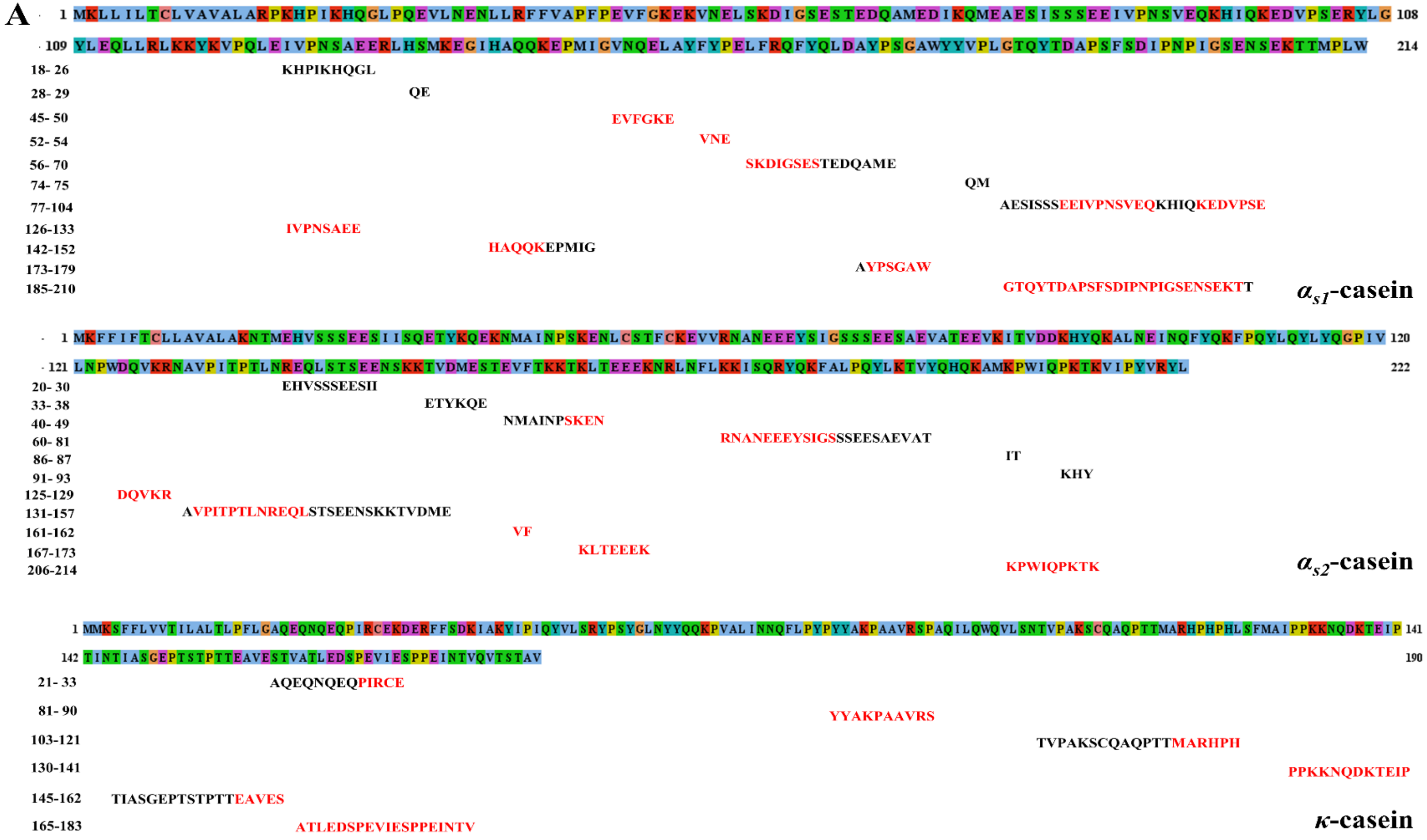

Figure S3. Continued

B

1 MKLLILTCLVAVALARPKHPIKHQGLPQEVLNENLLRFFVAPFPEVFGKEKVNELSKDIGSESTEDQAMEDIKQMEAESISSSEEIVPNSVEQKHIQKEDVPSERYLGYLEQLLRLKKYKVPQLEIVPNSAEERLHSMKEG 141

142 IHAQQKEPMIGVNQELAYFYPELFRQFYQLDAYPSGAWYYVPLGTQYTDAPSFSDIPNPIGSENSEKTTMPLW 214

18- 33 KHPINHRGLSPEVPNE

57-104 KDIGSESTEDQAMEDAKQMKAGSSSSSEEIVPNSAEQKYIQKEDVPSE

126-134 IVPNSAEER

136-148 HSMKEG- -IHAQQKE

173-177 AYPSG

186-210 TQYTDAPSFSDIPNPIGSENSGKTT

α

α_{s1}-casein

1 MKFFIFTCLLAVALAKHKMEHVSSSEEPINIFQEIYKQEKNMAIHPRKEKLCTTSCEEVVRNANEEEYSIRSSSEESAEVAPEEIKITVDDKHYQKALNEINQFYQKFPQYLQYPYQGPIVLNPWDQVKRNAGPFTPTVNREQLSTSEENSKKTI 155

156 DMESTEVFTKKTKLTEEEKNRLNFLKKISQYYQKFAWPQYLKTVDQHQKAMKPWTQPKTNAIPYVRYL 223

22- 29 VSSSEEPI

61- 84 RNANEEEYSIRSSSEESAEVAPEE

86- 87 KI

92- 94 KHY

113-117 QYPYQ

119-120 PI

126-158 DQVKRNAGPFTPTVNREQLSTSEENSKKTI- -DME

162-163 VF

168-174 KLTEEEK

201-217 QHQKAMKPWTQPKTNAI

α_{s2}-casein

1 MMKSFFLVVTILALTLPFLGAQEQNQEQPICCEKDERFFDDKIAKYIPIQYVLSRYPSYGLNYYQQRPVALINNQFLPYPYYAKPVAVRSPAQTLQWQVLPNTVPAKSCQDQPTTLARHPHPHLSFMAIPPKKDQDKTEVPAINTIASAEPTVH 15

155 STPTTEAIVNTVDNPEASSESIASASETNTAQVTSTEV 19

21- 32 AQEQNQEQPICC

37- 38 RF

81- 94 YYAKPVAVRSPAQT

102-119 NTVPAKSCQDQPTTLARH

130-142 PPKKDQDKTEVPA

146-192 IASAEPTVH- -STPTTEAIVNTVDNPEASSESIASASETNTAQVTSTEV

κ-casein

Figure S3. Continued

C

1 MKLLILTCLVAVALARPKYPLRYPEVFQNEPDSIEEVLNKRKILELAVVSPIQFRQENIDELKDTRNEPTEDHIMEDTERKESGSSSSEEVVSSTTEQKDILKEDMPSQRYLEELHRLNKYKLLQLEAIRDQKLIPRVKLSSHPYLEQLYRINE 154

155 DNHPQLGEPVKVVTQEQAYFHLEPFPQFFQLGASPYVAWYYPPQVMQYIAHPSSYDTPEGIASEDGGKTDVMPQWW 230

18- 22 KYPLR

26- 35 VFQNEPDSIE

59-110 IDELKDTRNEPTEDHIMEDTERKESGSSSSEEVVSSTTEQKDILKEDMPSQR

154-168 E- -DNHPQLGEPVKVVT

194-195 YY

205-226 HPSSYDTPEGIASEDGGKTDVM

α_{s1}-casein

1 MKFFIFTCLLAVVLAKHEMDQGSSSEESINVSQQKFKQVKKVAIHPSKEDICSTFCEEAVRNIKEVESAEVPTENKISQFYQKWKFLQYLQALHQGQIVMNPWDQGKTRAYPFIPTVNTEQLSISEESTEVPTEESTEVFTKKTELTEEEKDHQ 154

155 KFLNKIYQYYQTFLWPEYLKTVYQYQKTMTPWNHIKRYF 193

19- 31 MDQGSSSEESINV

44- 50 IHPSKED

65- 77 EVESAEVPTENKI

103-110 WDQGKTRA

116-119 TVNT

122-141 LSISEESTEVPTEESTEVFT

144-153 TELTEEEKDH

181-184 KTMT

α_{s2}-casein

1 MKSFFLVVTILALTLPFLGAEVQNQEQPTCFEKVERLLNEKTVKYFPIQFVQSRYPSYGINYYQHRLAVPINNQFIPYPNYAKPVAIRLHAQIPQCQALPNIDPPTVERRPRPRPSFIAIPPKKTQDKTVNPAINTVATVEPPVIPTAEPAVNT 154

155 VVIAEASSEFITTSTPETTTVQITSTEI 182

22- 30 VQNQEQPTC

55- 58 YPSY

76- 83 IPYPNYAK

98-135 ALPNIDPPTVERRPRPRPSFIAIPPKKTQDKTVNPAIN

137-152 VATVEPPVIPTAEPAV

163-177 EFITTSTPETTTVQI

κ-casein

Figure S3. Continued

D

1 MKVFILACLVAVALARLKLPHRQPEIIQNEQDSREKVLKERKFPSFALEYINELNRQRELLKEKQKDEHKDTSNESTEEYLIEDPEQQESSSTSSSEEVVPINTEKRI 108

109 PREDMLYQHTLEQLRRLSKYNQLQLQAIHAQRKPMRVVNQEQAYFYLEPFQPSYQLDVYPYAAWFHPAQIMQHVAYSPFHDTAKLIASENSEKTDIIPEW 208

21-22 HR

24-36 PEIIQNEQDSREK

63-109 EKQKDEHKDTSNESTEEYLIEDPEQQESSSTSSSEEVVPINTEKRI-P

158-163 FQPSYQ

186-188 PFH

196-203 SENSEKTD

***α*~s1~-casein**

1 MKFFIFTCLLAVALAKHNMEHRSSSEDSVNISQEKFKQEKYVVIPTSKESICSTSCEEATRNINEMESAKFPTEVYSSSSSSEESAKFPTEREEKEVEEKHHLKQLNKINQFYEKLNFLQYLQALRQPRIVLTPWDQTKTGDSPFIPIVNTE 152

153 QLFTSEEIPKKTVDMESTEVVTEKTELTEEEKNYLKLLYYEKFTLPQYFKIVRQHQTTMDPRSHRKTNSYQIIPVLRYF 231

20-31 EHRSSSEDSVNI

34-37 EKFK

46-51 TSKESI

56-66 CEEATRNINEM

68-98 SAKFPTEVYSSSSSSEESAKFPTEREEKEVE

135-145 WDQTKTGDSPF

159-168 EIPKKTVDME

170-184 TEVVTEKTELTEEEK

208-220 QTTMDPRSHRKTN

α_{s2}-casein

1 MKSFFLVVNILALTLPFLGAEVQNQEQPTCHKNDERFFDLKTVKYIPIYYVLNSSPRYEPIYYQHRLALLINNQHMPYQYYARPAAVRPHVQIPQWQVLPNIYPSTVVRHPCPHPSFIAIPPKKLQEITVIPKINTIATVEPTPIPTPEPTVNN 154

155 AVIPDASSEFIIASTPETTTVPVTSPVVQKL 185

22-33 VQNQEQPTCHKN

55-60 SPRYEP

80-88 YYARPAAVR

108-115 VRHPCPHP

119-120 AI

123-125 KKL

138-160 ATVEPTPIPTPEPTVNN-AVIPDA

169-180 TPETTTVPVTSP

***κ*-casein**

Figure S4. Predicting results of distribution of secondary structure in allergenic subtypes of CM, GM, CAM and MM. Panels A to D represent CM, GM, CAM and MM, respectively.

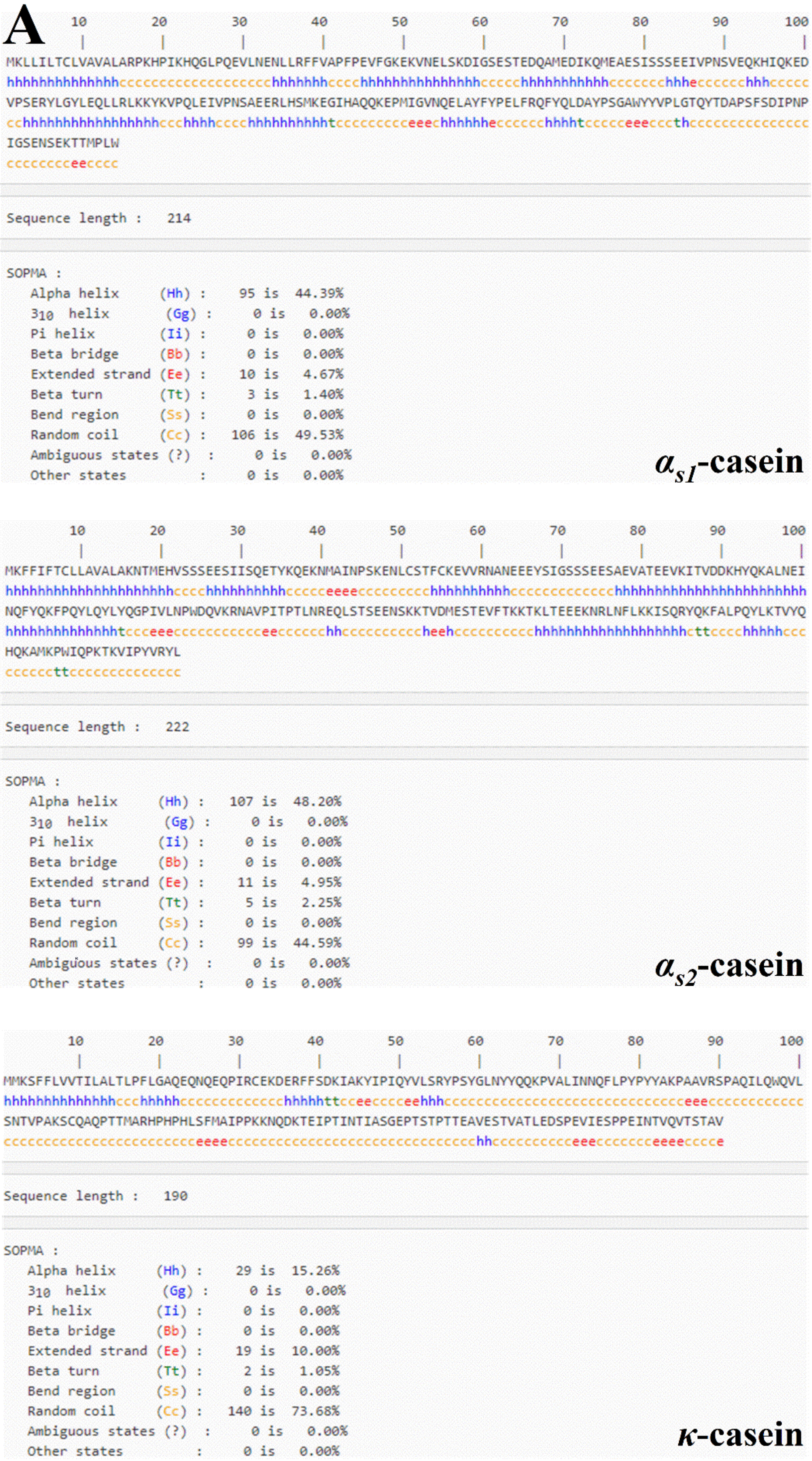

Figure S4. Continued

B

```
        10        20        30        40        50        60        70        80        90       100
         |         |         |         |         |         |         |         |         |         |
MKLLILTCLVAVALARPKHPINHRGLSPEVPNENLLRFVVAPFPEVFRKENINELSKDIGSESTEDQAMEDAKQMKAGSSSSSEEIVPNSAEQKYIQKED
hhhhhhhhhhhhhhcccccccccccccccccchhhhhhhhcccchhhhhhhhhhhhhhhccccchhhhhhhhhhccccccccchhecccccchhhhhhhcc
VPSERYLGYLEQLLRLKKYNVPQLEIVPKSAEEQLHSMKEGNPAHQKQPMIAVNQELAYFYPQLFRQFYQLDAYPSGAWYYLPLGTQYTDAPSFSDIPNP
ccchhhhhhhhhhhhhhhcccchhhhcccchhhhhhhhhttcccccccccceecchtchheeecccchhheetccctteeeeccttcccccccccccccccc
IGSENSGKTTMPLW
cccccccceeeccc

Sequence length :   214

SOPMA :
   Alpha helix      (Hh) :    90 is  42.06%
   310  helix       (Gg) :     0 is   0.00%
   Pi helix         (Ii) :     0 is   0.00%
   Beta bridge      (Bb) :     0 is   0.00%
   Extended strand  (Ee) :    15 is   7.01%
   Beta turn        (Tt) :     7 is   3.27%
   Bend region      (Ss) :     0 is   0.00%
   Random coil      (Cc) :   102 is  47.66%
   Ambiguous states (?)  :     0 is   0.00%
   Other states          :     0 is   0.00%
```

α_{s1}-casein

```
        10        20        30        40        50        60        70        80        90       100
         |         |         |         |         |         |         |         |         |         |
MKFFIFTCLLAVALAKHKMEHVSSSEEPINIFQEIYKQEKNMAIHPRKEKLCTTSCEEVVRNANEEEYSIRSSSEESAEVAPEEIKITVDDKHYQKALNE
hhhhhhhhhhhhhhhhhhhhhcccchhhhhhhhhhhcttcceeeeccccccchhhhhhhhhcchhhhhccccccccccccccchhhhhhhhhhhhhhhhh
INQFYQKFPQYLQYPYQGPIVLNPWDQVKRNAGPFTPTVNREQLSTSEENSKKTIDMESTEVFTKKTKLTEEEKNRLNFLKKISQYYQKFAWPQYLKTVD
hhhhhhcchhhhhhhhtccceeccccccccccccccccccccccccchhccccceeeecccccccccchhhhhhhhhhhhhhhhhhhcttcccchhhhhhc
QHQKAMKPWTQPKTNAIPYVRYL
chhhccctteeeeeeeeccccccc

Sequence length :   223

SOPMA :
   Alpha helix      (Hh) :   106 is  47.53%
   310  helix       (Gg) :     0 is   0.00%
   Pi helix         (Ii) :     0 is   0.00%
   Beta bridge      (Bb) :     0 is   0.00%
   Extended strand  (Ee) :    16 is   7.17%
   Beta turn        (Tt) :     7 is   3.14%
   Bend region      (Ss) :     0 is   0.00%
   Random coil      (Cc) :    94 is  42.15%
   Ambiguous states (?)  :     0 is   0.00%
   Other states          :     0 is   0.00%
```

α_{s2}-casein

```
        10        20        30        40        50        60        70        80        90       100
         |         |         |         |         |         |         |         |         |         |
MMKSFFLVVTILALTLPFLGAQEQNQEQPICCEKDERFFDDKIAKYIPIQYVLSRYPSYGLNYYQQRPVALINNQFLPYPYYAKPVAVRSPAQTLQWQVL
hhhhhhhhhhhhhhccchhhhhccccccccccccchhhhhttcceecccceehhhcccccccccccccccccccccccccccccceeecccccccccccc
PNTVPAKSCQDQPTTLARHPHPHLSFMAIPPKKDQDKTEVPAINTIASAEPTVHSTPTTEAIVNTVDNPEASSESIASASETNTAQVTSTEV
ccccccccccccccccccccccccceeecccccccccccccccceeecccccccccccccceeeecccccccchhhhhhcccccchheeeehhh

Sequence length :   192

SOPMA :
   Alpha helix      (Hh) :    37 is  19.27%
   310  helix       (Gg) :     0 is   0.00%
   Pi helix         (Ii) :     0 is   0.00%
   Beta bridge      (Bb) :     0 is   0.00%
   Extended strand  (Ee) :    21 is  10.94%
   Beta turn        (Tt) :     2 is   1.04%
   Bend region      (Ss) :     0 is   0.00%
   Random coil      (Cc) :   132 is  68.75%
   Ambiguous states (?)  :     0 is   0.00%
   Other states          :     0 is   0.00%
```

κ-casein

Figure S4. Continued

C

```
        10         20         30         40         50         60         70         80         90        100
         |          |          |          |          |          |          |          |          |          |
MKLLILTCLVAVALARPKYPLRYPEVFQNEPDSIEEVLNKRKILELAVVSPIQFRQENIDELKDTRNEPTEDHIMEDTERKESGSSSSEEVVSSTTEQKD
hhhhhhhhhhhhhhccccccccchhhhcccccchhhhhhhhhhhhhheeccchhhhhhhhhhhhhhhccchhhhhhhhhhhhhhcccchheecccccchh
ILKEDMPSQRYLEELHRLNKYKLLQLEAIRDQKLIPRVKLSSHPYLEQLYRINEDNHPQLGEPVKVVTQEQAYFHLEPFPQFFQLGASPYVAWYYPPQVM
cchhhcchhhhhhhhhhhtcchhhhhhhhhhtccchheecccchhhhhhhhhhhttcccccccchheeechhhhhhecccccchhhhhtcccceeeeccttthh
QYIAHPSSYDTPEGIASEDGGKTDVMPQWW
ccccccccccccccccccccccceeeccch

Sequence length :   230

SOPMA :
   Alpha helix      (Hh) :   116 is  50.43%
   310  helix       (Gg) :     0 is   0.00%
   Pi helix         (Ii) :     0 is   0.00%
   Beta bridge      (Bb) :     0 is   0.00%
   Extended strand  (Ee) :    17 is   7.39%
   Beta turn        (Tt) :     7 is   3.04%
   Bend region      (Ss) :     0 is   0.00%
   Random coil      (Cc) :    90 is  39.13%
   Ambiguous states (?)  :     0 is   0.00%
   Other states          :     0 is   0.00%
```

α_{s1}**-casein**

```
        10         20         30         40         50         60         70         80         90        100
         |          |          |          |          |          |          |          |          |          |
MKFFIFTCLLAVVLAKHEMDQGSSSEESINVSQQKFKQVKKVAIHPSKEDICSTFCEEAVRNIKEVESAEVPTENKISQFYQKWKFLQYLQALHQGQIVM
hhhhhhhhhhhhhhhhhhhhhcccchhhhhhhhhhhcttcceeeecccccccccccccchhhhhhhhhhhhhhhhhhhhhhhhhhhhhhhhhhhcccheee
NPWDQGKTRAYPFIPTVNTEQLSISEESTEVPTEESTEVFTKKTELTEEEKDHQKFLNKIYQYYQTFLWPEYLKTVYQYQKTMTPWNHIKRYF
ccccccccccccccchhhhhhcccchhhccccccccceeehccchhhhhhhhhhhhhhhhhhhhhhccccchhhhhhhhhhhhccttchhhhhh

Sequence length :   193

SOPMA :
   Alpha helix      (Hh) :   116 is  60.10%
   310  helix       (Gg) :     0 is   0.00%
   Pi helix         (Ii) :     0 is   0.00%
   Beta bridge      (Bb) :     0 is   0.00%
   Extended strand  (Ee) :    10 is   5.18%
   Beta turn        (Tt) :     4 is   2.07%
   Bend region      (Ss) :     0 is   0.00%
   Random coil      (Cc) :    63 is  32.64%
   Ambiguous states (?)  :     0 is   0.00%
   Other states          :     0 is   0.00%
```

α_{s2}**-casein**

```
        10         20         30         40         50         60         70         80         90        100
         |          |          |          |          |          |          |          |          |          |
MKSFFLVVTILALTLPFLGAEVQNQEQPTCFEKVERLLNEKTVKYFPIQFVQSRYPSYGINYYQHRLAVPINNQFIPYPNYAKPVAIRLHAQIPQCQALP
hhhhhhhhhhhhhccchhhhhcccccccccccchhhhhhttcccccceeeehcccccccccceeccccccccccccccccccccceeeccccccccccccc
NIDPPTVERRPRPRPSFIAIPPKKTQDKTVNPAINTVATVEPPVIPTAEPAVNTVVIAEASSEFITTSTPETTTVQITSTEI
ccccccccccccccccceeeccccccccccccccceeecccccccccccccceeeeeccccceeecccccccceeeeccccce

Sequence length :   182

SOPMA :
   Alpha helix      (Hh) :    25 is  13.74%
   310  helix       (Gg) :     0 is   0.00%
   Pi helix         (Ii) :     0 is   0.00%
   Beta bridge      (Bb) :     0 is   0.00%
   Extended strand  (Ee) :    28 is  15.38%
   Beta turn        (Tt) :     2 is   1.10%
   Bend region      (Ss) :     0 is   0.00%
   Random coil      (Cc) :   127 is  69.78%
   Ambiguous states (?)  :     0 is   0.00%
   Other states          :     0 is   0.00%
```

κ-casein

Figure S4. Continued

D

```
        10        20        30        40        50        60        70        80        90       100
         |         |         |         |         |         |         |         |         |         |
MKVFILACLVAVALARLKLPHRQPEIIQNEQDSREKVLKERKFPSFALEYINELNRQRELLKEKQKDEHKDTSNESTEEYLIEDPEQQESSSTSSSEEVV
hhhhhhhhhhhhhhccttccccchheecccccchhhhhhhttchhhhhhhhhhhhhhhhhhhhhhhhhhhccccccchhhhhhccccccccccccchhec
PINTEKRIPREDMLYQHTLEQLRRLSKYNQLQLQAIHAQRKPMRVVNQEQAYFYLEPFQPSYQLDVYPYAAWFHPAQIMQHVAYSPFHDTAKLIASENSE
cccccccccccccchhhhhhhhhhhhhcchhhhhhhhhhhhhhhhhhtcchhheecccchhhhhtccccceeeccttthhheecccccccccccccccccc
KTDIIPEW
ccceeccc

Sequence length :   208

SOPMA :
   Alpha helix     (Hh) :   100 is  48.08%
   310  helix      (Gg) :     0 is   0.00%
   Pi helix        (Ii) :     0 is   0.00%
   Beta bridge     (Bb) :     0 is   0.00%
   Extended strand (Ee) :    12 is   5.77%
   Beta turn       (Tt) :     8 is   3.85%
   Bend region     (Ss) :     0 is   0.00%
   Random coil     (Cc) :    88 is  42.31%
   Ambiguous states (?) :     0 is   0.00%
   Other states         :     0 is   0.00%
```

α_{s1}-casein

```
        10        20        30        40        50        60        70        80        90       100
         |         |         |         |         |         |         |         |         |         |
MKFFIFTCLLAVALAKHNMEHRSSSEDSVNISQEKFKQEKYVVIPTSKESICSTSCEEATRNINEMESAKFPTEVYSSSSSSEESAKFPTEREEKEVEEK
hhhhhhhhhhhhhhhhhhhcccccchhhhhhhhhhhcttceeeeecccccccccchhhhhhhhhhhhhcccchhhhhhccccccccccccccccchhhhh
HHLKQLNKINQFYEKLNFLQYLQALRQPRIVLTPWDQTKTGDSPFIPIVNTEQLFTSEEIPKKTVDMESTEVVTEKTELTEEEKNYLKLLYYEKFTLPQY
hhhhhhhhhhhhhhhhhhhhhhhhccccceeeccccccccccccccccccchhcccccccccccceecccccccchhhhhhhhhhhhhhhhhccccccchh
FKIVRQHQTTMDPRSHRKTNSYQIIPVLRYF
hhhcccccccccccccccccceeecceechh

Sequence length :   231

SOPMA :
   Alpha helix     (Hh) :   104 is  45.02%
   310  helix      (Gg) :     0 is   0.00%
   Pi helix        (Ii) :     0 is   0.00%
   Beta bridge     (Bb) :     0 is   0.00%
   Extended strand (Ee) :    15 is   6.49%
   Beta turn       (Tt) :     2 is   0.87%
   Bend region     (Ss) :     0 is   0.00%
   Random coil     (Cc) :   110 is  47.62%
   Ambiguous states (?) :     0 is   0.00%
   Other states         :     0 is   0.00%
```

α_{s2}-casein

```
        10        20        30        40        50        60        70        80        90       100
         |         |         |         |         |         |         |         |         |         |
MKSFFLVVNILALTLPFLGAEVQNQEQPTCHKNDERFFDLKTVKYIPIYYVLNSSPRYEPIYYQHRLALLINNQHMPYQYYARPAAVRPHVQIPQWQVLP
hhhhhhhhhhhhhccchhhhhhcccccccccccchhhhhttccccccceeeecccccccccccccccceecccccccccccccceeeccccccccccccc
NIYPSTVVRHPCPHPSFIAIPPKKLQEITVIPKINTIATVEPTPIPTPEPTVNNAVIPDASSEFIIASTPETTTVPVTSPVVQKL
cccccccccccccccceeeccccccccccccccceeecccccccccccceecccccccccheeeeecccccceeeeccceeccc

Sequence length :   185

SOPMA :
   Alpha helix     (Hh) :    25 is  13.51%
   310  helix      (Gg) :     0 is   0.00%
   Pi helix        (Ii) :     0 is   0.00%
   Beta bridge     (Bb) :     0 is   0.00%
   Extended strand (Ee) :    28 is  15.14%
   Beta turn       (Tt) :     2 is   1.08%
   Bend region     (Ss) :     0 is   0.00%
   Random coil     (Cc) :   130 is  70.27%
   Ambiguous states (?) :     0 is   0.00%
   Other states         :     0 is   0.00%
```

κ-casein
